# Catalase deficiency facilitates the shuttling of free fatty acid to brown adipose tissue through lipolysis mediated by ROS during sustained fasting

**DOI:** 10.1186/s13578-021-00710-5

**Published:** 2021-12-07

**Authors:** Raghbendra Kumar Dutta, Joon No Lee, Yunash Maharjan, Channy Park, Seong-Kyu Choe, Ye-Shih Ho, Raekil Park

**Affiliations:** 1 Department of Biomedical Science & Engineering, GRI, Gwangju Institute of Science & Technology, Gwangju, 61005 Republic of Korea; 2 Department of Microbiology and Center for Metabolic Function Regulation, Wonkwang University School of Medicine, Iksan, Jeonbuk 54538 Republic of Korea; 3 Institute of Environmental Health Sciences and Department of Biochemistry and Molecular Biology, Wayne State University, Detroit, MI 48201 USA

**Keywords:** Catalase, Sustained fasting, Reactive Oxygen species (ROS), Lipolysis, Thermogenesis

## Abstract

**Background:**

Fatty acids (FA) derived from adipose tissue and liver serve as the main fuel in thermogenesis of brown adipose tissue (BAT). Catalase, a peroxisomal enzyme, plays an important role in maintaining intracellular redox homeostasis by decomposing hydrogen peroxide to either water or oxygen that oxidize and provide fuel for cellular metabolism. Although the antioxidant enzymatic activity of catalase is well known, its role in the metabolism and maintenance of energy homeostasis has not yet been revealed. The present study investigated the role of catalase in lipid metabolism and thermogenesis during nutrient deprivation in catalase-knockout (KO) mice.

**Results:**

We found that hepatic triglyceride accumulation in KO mice decreased during sustained fasting due to lipolysis through reactive oxygen species (ROS) generation in adipocytes. Furthermore, the free FA released from lipolysis were shuttled to BAT through the activation of CD36 and catabolized by lipoprotein lipase in KO mice during sustained fasting. Although the exact mechanism for the activation of the FA receptor enzyme, CD36 in BAT is still unclear, we found that ROS generation in adipocytes mediated the shuttling of FA to BAT.

**Conclusions:**

Taken together, our findings uncover the novel role of catalase in lipid metabolism and thermogenesis in BAT, which may be useful in understanding metabolic dysfunction.

**Graphical Abstract:**

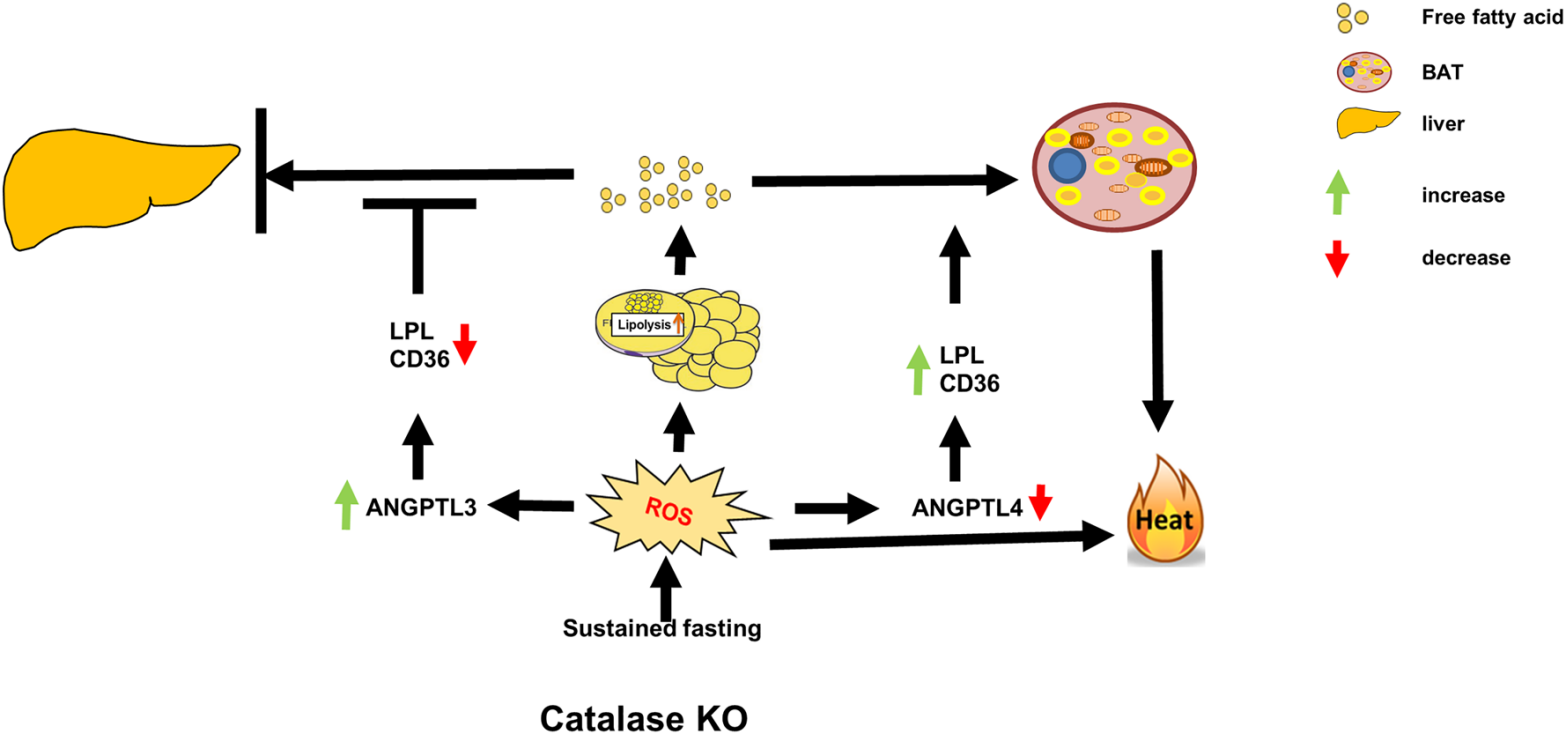

**Supplementary Information:**

The online version contains supplementary material available at 10.1186/s13578-021-00710-5.

## Background

Adipose tissue consists of white adipose tissue (WAT), which stores energy in the form of triacylglycerol (TG), and brown adipose tissue (BAT) which dissipates energy in the form of heat. BAT differs from WAT as it contains small and multilocular lipid droplets (LDs) and abundant mitochondria containing uncoupling protein 1 (UCP1), which is used for dissipation of energy in the form of heat [1]. The main fuel for BAT thermogenesis is the oxidation of fatty acids (FA) derived from WAT through lipolysis or through VLDL and acyl carnitines produced in the liver [2]. During stress or nutrient-deprivation, TG are the major form of lipids in the adipose tissue, where lipolysis takes place and induces the release free fatty acids (FFA) and glycerol into the circulation and mobilized into the liver, where it induces excessive β-oxidation for energy homeostasis [3–5]. The enzyme lipoprotein lipase (LPL) catalyzes the hydrolysis of TG to generate free fatty acids (FFAs). During fasting, lipolysis is induced in WAT through regulation of angiopoietin-like protein 4 (ANGPTL4), which galvanizes LPL activity and thereby directs circulating TG to the liver [6]. ANGPTLs, are a family of proteins structurally similar to angiopoietins, that play an important role in lipid metabolism, inflammation and cancer [7]; Among ANGPTLs, ANGPTL3, ANGPTL4, and ANGPTL8 play major roles in the trafficking and metabolism of lipid by suppressing LPL activity [3, 8]. Although both ANGPTL3 and ANGPTL4 act as an important regulators of LPL-mediated FA uptake, their expression in tissues varies. ANGPTL3 is a circulating protein synthesized exclusively in the liver [8–10], whereas ANGPTL4 is synthesized in adipose tissue (AT) [11, 12]. Hence, to fuel BAT thermogenesis, the FA transporter enzyme and LPL must be activated.

It is well documented that reactive oxygen species (ROS) regulate lipolysis and BAT thermogenesis [13–16]. ROS are generated during metabolism mainly in the mitochondria and peroxisomes, where oxygen consumption is high [17, 18]. High levels of ROS exert toxic effects on cells, causing the accumulation of oxidative damage in diverse cellular locations [18]. However, the enzyme catalase in the peroxisomes limit this damaging effect by detoxifying H_2_O_2_ to either water or oxidizing other organic compounds that provide a major source of metabolic energy for cell survival. During β-oxidation, several H_2_O_2_ producing oxidases generated in peroxisomes are converted to hydroxyl radicals (•OH), making peroxisomal membranes vulnerable to lipid peroxidation [18]. Although catalase is important for ROS detoxification, its deficiency in mice and humans is phenotypically normal [19, 20]. Recently, we showed that catalase depletion during starvation causes selective pexophagy in the liver through ROS accumulation in vivo and in vitro [21, 22], while overexpression of catalase has been shown to be beneficial [23–26]. Although catalase is well described as an antioxidant enzyme, its role in maintaining energy homeostasis during starvation has not yet been elucidated.

In this study, we aimed to investigate the role of catalase in the contribution of energy metabolism under fasting conditions. Both wild-type (WT) and catalase-knockout (KO) mice were subjected to sustained fasting for 48 h to ensure sufficient generation of ROS. Our study showed that lipolysis was induced in KO mice through ROS generation, which deviates FFAs to BAT for thermogenesis during sustained fasting.

## Results

### Catalase deficiency markedly decreased the hepatic lipid accumulation during sustained fasting

Starvation produces a complex array of adaptive metabolic responses that activate β-oxidation in both peroxisomes and mitochondria [27, 28]. To investigate the metabolic response of peroxisomes to starvation, mice were fasted for different time intervals (0, 6, 12, 24, 36, and 48 h) and immunoblot analysis was performed using liver homogenates to examine the differential expression of peroxisome markers. Among the peroxisome proteins, only catalase was significantly increased in a time-dependent manner after fasting. The expression levels of other peroxisome markers, including ACOX1, DBP, PEX5, and PMP70, did not change during different time intervals of fasting (Additional file 1: Fig. S1A). In addition, there were no significant changes in the protein expression levels of other antioxidants, such as GPX, PRXIII, SOD1 and SOD2. Consistently, the expression level of catalase mRNA significantly increased during sustained fasting, whereas transcriptional activation of other genes, including peroxisomes and antioxidants, remained at a consistent level similar to the protein levels (Additional file 1: Fig. S1B). Catalase activity was also measured from the liver homogenates of mice fasted at specific time intervals. The catalase activity significantly increased at 24 h of fasting and they goes up until sustained fasting (Additional file 1: Fig. S1C). Taken together, these data suggest that catalase might be a key player in liver metabolism during sustained fasting.

**Fig. 1:**
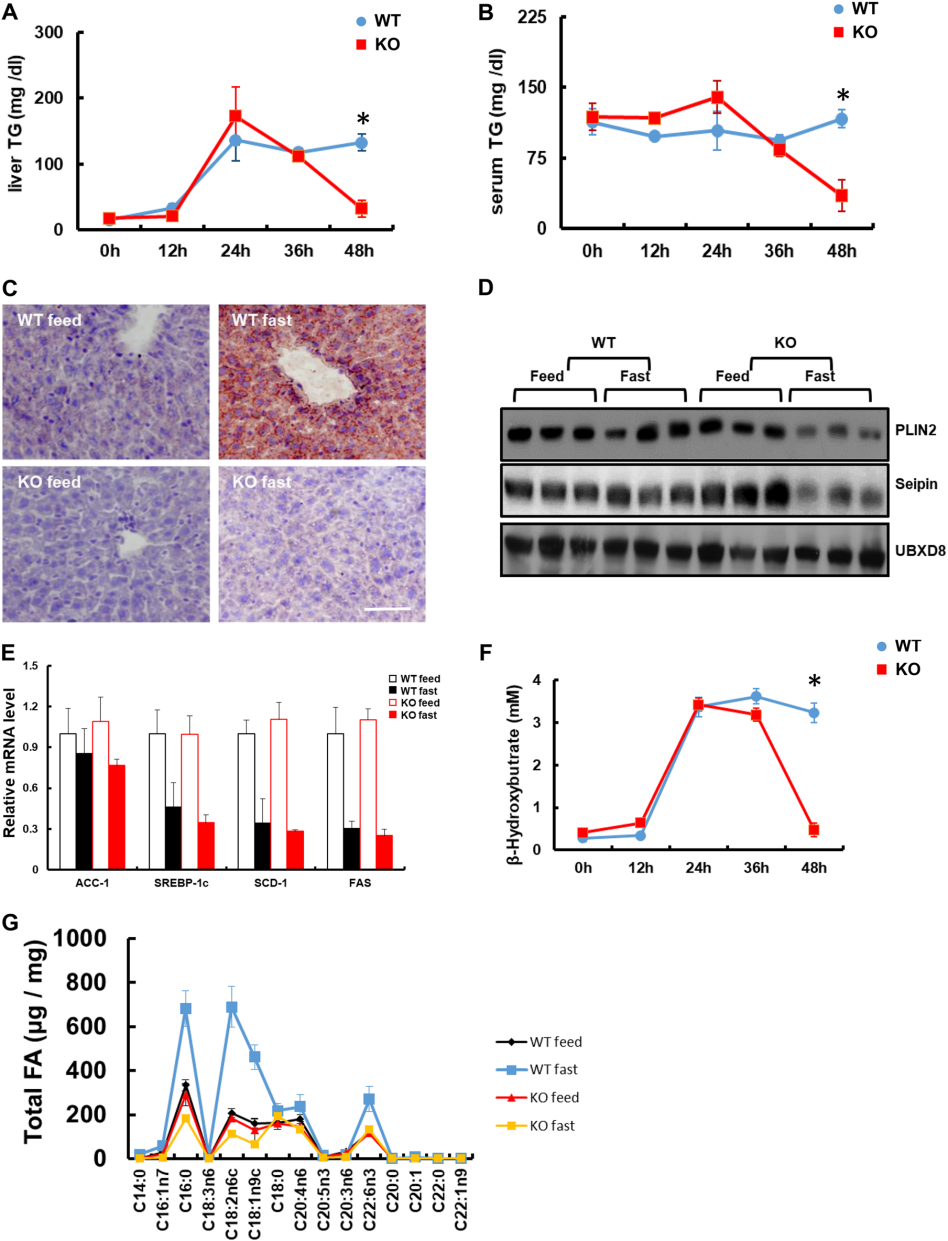
Catalase deficiency decreases the accumulation of lipid in liver during sustained fasting. Liver samples from mice fasted at the indicated time were homogenized, and TG levels were analyzed from **A** liver and **B** serum (n = 6). **C** Liver tissues from mice cryosectioned and stained with ORO. Scale bar represents 20 μm. **D** Immunoblot analysis of liver tissue. Protein expression using whole-cell lysates with the indicated antibodies (n = 3). **E** qPCR analysis of FA metabolism-related genes from the liver of mice fed and fasted for 48 h. **F** β-hydroxybutyrate was analyzed from the serum of mice fasted for indicated time. **G** GC–MS for total FA of long Chain and VLCFA from liver samples (n = 3). Values represent mean ± SD (n = 3, 4). *P < 0.001 WT 48 h fasting versus KO 48 h of fasting

To determine whether catalase expression might have been affected by nutrient status, catalase-KO and WT mice were subjected to 48 h of starvation, represented as sustained fasting. Catalase deficiency was confirmed by immunoblot and immunofluorescence analyses from the liver tissues (Additional file 1: Fig. S2A, B). It is well known that starvation induces the hydrolysis of TG stored in adipose tissues, releasing FFAs in the plasma. These are taken up by the liver for TG formation, β-oxidation, and VLDL secretion for energy supply [29, 30]. Biochemical analysis was performed to examine TG levels in the liver and serum after fasting for different time intervals (0, 12, 24, 36, and 48 h). Hepatic TG of WT mice started to increase at early hours of fasting, reached a peak at 24 h, and remained sustained until 48 h. However, it drastically decreased after fasting for 24 h and returned to the resting status level at 48 h in KO mice (Fig. 1A). Similarly, TG in the serum was markedly decreased in KO mice after 24 h of fasting, whereas it was sustained in WT mice (Fig. 1B). To evaluate lipid accumulation in the liver during sustained fasting, Oil Red O (ORO) staining was performed (Fig. 1C). ORO staining, shown as a red signal, indicated lipid accumulation in the liver of WT mice during sustained fasting, whereas it was clearly absent in KO mice (Fig. 1C). Consistently, immunoblot analysis for the lipid droplet-associated protein perilipin 2 (PLIN2) and the lipid biogenesis protein seipin, revealed that increased lipogenesis and accumulation of lipid droplets occurred only in the liver of WT mice, but not in KO mice (Fig. 1D). To address the metabolic response to sustained fasting, we analyzed the expression of acetyl coenzyme A carboxylase 1 (ACC1), which is involved in FA synthesis. The mRNA expression of *ACC1*, decreased after sustained fasting in both WT and KO mice; however, there was no significant difference between the two experimental groups (Fig. 1E). *SREBP1C* mRNA, involved in the induction of lipogenesis that facilitates storage of fatty acids as triglycerides, decreased significantly after sustained fasting without significant difference between the two experimental groups (Fig. 1E). Similarly, SCD-1(Stearoyl-CoA desaturase-1), a key enzyme for fatty acid metabolism responsible for forming a monounsaturated fatty acid. And FAS (fatty acid synthase) catalyzes the de novo biosynthesis of long-chain saturated fatty acids were significantly decreased after sustained fasting without any significant difference between the two experimental groups. We also measured β-hydroxybutyrate levels in the liver after sustained fasting for 48 h (Fig. 1F); the levels started to increase at 12 h, reached a maximum at approximately 24 h, and sustained for a further 48 h in WT mice. However, it drastically dropped to the basal level at 48 h in KO mice. To assess total FA, gas chromatography-mass spectrometry (GC–MS) was performed (Fig. 1G). Accumulation of many FA species, including both long chain and median chain FA, decreased significantly during sustained fasting in KO mice compared to WT mice (Fig. 1G). Taken together, these data suggest that catalase deficiency resulted in decreased lipid accumulation in the liver during sustained fasting.

### Catalase deficiency during sustained fasting accelerated lipolysis from the white adipose tissue through ROS generation

As hepatic TG levels were diminished in KO mice during sustained fasting, we reasoned that TG consumption might be induced by the activation of FA oxidation (FAO) in the liver of fasted KO mice. Therefore, anomalies or extreme activation of mitochondrial or peroxisomal FAO could be the prime reason for the decreased hepatic TG accumulation in fasted KO mice. However, we did not find any involvement of mitochondrial or peroxisomal FAO [22]. Mitochondrial FAO did not change in either WT or KO mice, whereas peroxisome was decreased through autophagic degradation in KO mice during sustained fasting [22], indicating that TG depletion in the liver is not related to mitochondrial and peroxisomal FAO.

**Fig. 2:**
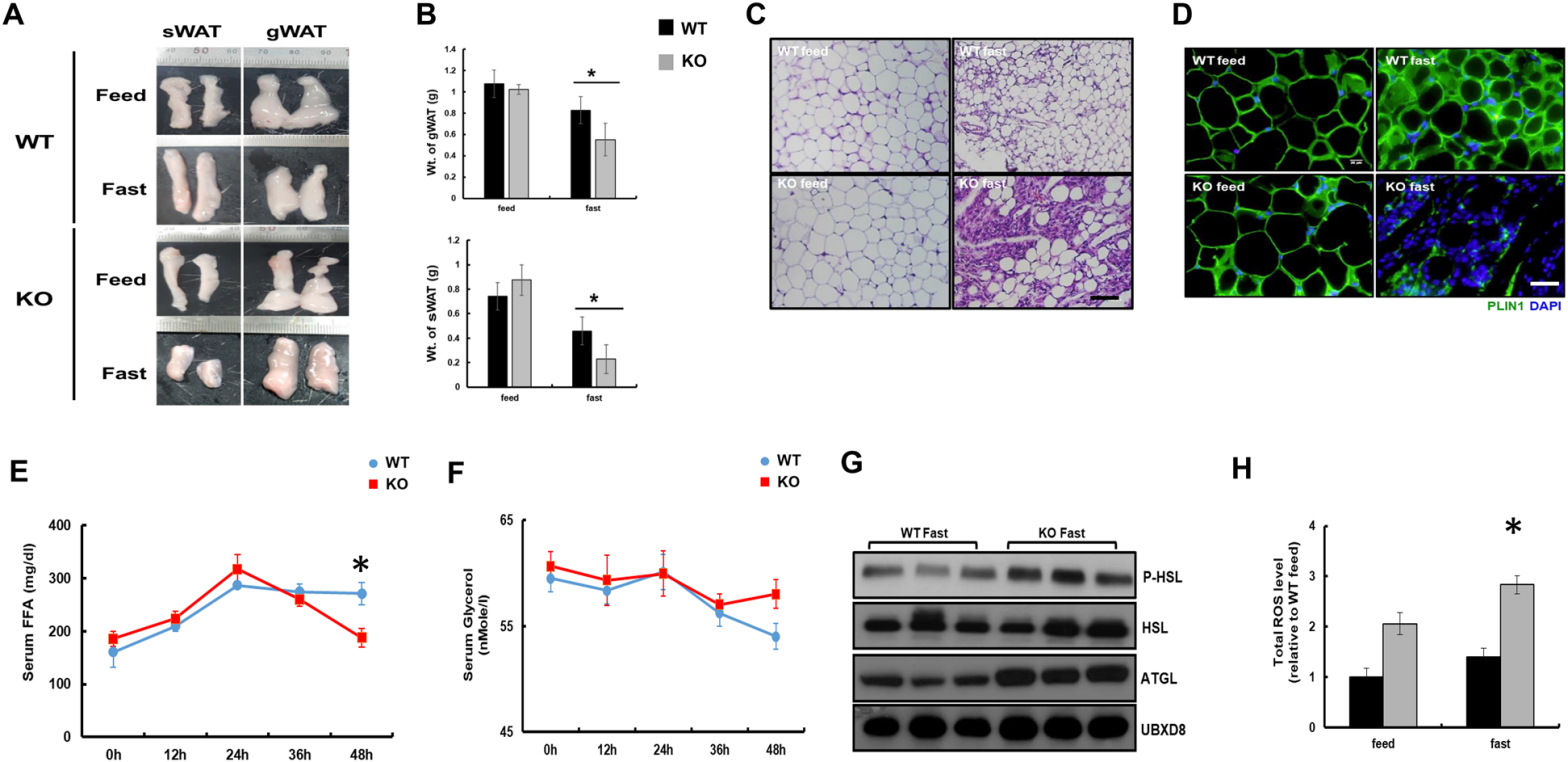
Catalase deficiency during sustained fasting decreases the fat mass that augmented lipolysis in WAT. **A** Images of subcutaneous and gonadal (sWAT, gWAT) white adipose tissuefrom mice (n = 6). **B** Analytical weight of gWAT and sWAT in gram (g) **C** Representative H&E staining of WAT from mice. **D** Representative fluorescence images of WAT, fixed and immunostained with anti-PLIN1 (green) and DAPI (blue).Scale bar represents 20 μm. **E** FFA and **F** glycerol level from mice serum fasted for indicated time (n = 6). **G** Proteins were extracted from WAT and immunoblot analysis was performed using whole-cell lysates with the indicated antibodies (n = 3). **H** Total ROS was measured from WAT. Values represent mean ± SD (n = 3, 4). *P < 0.05 WT 48 h fasting versus KO 48 h of fasting

Fasting is a unique stress condition that enhances lipid catabolism. Prolonged fasting is known to induce TG hydrolysis in adipose tissue, increasing the plasma concentration of FFAs and inducing hepatic TG accumulation [31, 32]. Hence, we measured serum FFAs levels and found that it decreased in KO mice during sustained fasting (Additional file 1: Fig. S3). Therefore, we assumed that KO mice might consume FFAs released from adipocytes faster than WT mice during sustained fasting. To examine this possibility, subcutaneous and gonadal white adipose tissue (sWAT and gWAT) from WT and KO mice was isolated and weighed. As expected, fat masses of KO mice were drastically reduced during sustained fasting compared to WT mice (Fig. 2A, B). Morphological analysis of adipose tissue sections by hematoxylin and eosin (H&E) staining showed bright eosinophilic cytoplasm with abundant lipid droplets in fed WT mice, whereas that of fasted WT mice were small in size with shrinkage of adipocytes and scanty eosinophilic cytoplasm. Similarly, the adipose tissue in fed KO mice was homogenous and uninucleate with nearly white-colored lipid droplets, while fasted KO mice showed a marked loss of adipocytes and an extended eosinophilic cytoplasm (Fig. 2C). We hypothesized, variable size in adipose tissue might exhibit inflammation that might lead to progressive lipodystrophy [33]. We analyzed the mRNA level of inflammatory cytokines in WT and KO adipocytes during fasting conditions. However we did not observe any change in mRNA level of cytokines such as TNFa, IL-6 and IL-1b (Additional file 1: Fig S4A). We also confirmed the adipocytes death by TUNEL (Terminal deoxynucleotidyl transferase dUTP nick end labeling) assay, where we did not observe any TUNEL positive induction in KO adipocytes fasted for 48 h (Additional file 1: Fig. S4B). We also did not find any changes in protein expression of MAC-2, a marker for macrophage induction in adipocytes of WT and catalase KO mice during sustained fasting (Additional file 1: Fig. S4C). Immunofluorescence staining of PLIN1, a lipid droplet-associated protein found in adipose tissue, had markedly decreased only in fasted KO mice (Fig. 2D). We also check the protein level of lipid droplet biogenesis protein seipin and PLIN1 by immunoblot in adipocytes. The protein expression of both seipin and PLIN1 was decreased in KO adipocytes during sustained fasting (Additional file 1: Fig S4C). As fat mass in adipose tissue was drastically decreased and lipogenesis protein including PLIN1 and seipin was decreased, we hypothesized that induction of lipolysis in WAT could be one of the reasons for the depletion of fat mass observed during sustained fasting in KO mice. Thus, we measured the serum FFAs and glycerol levels at different time intervals (0, 12, 24, 36, and 48 h) in WT and KO mice. After 36 h of fasting, FFAs level increased in a time-dependent manner and then remained stable in WT mice, while the level had decreased slightly in KO mice after 36 h of fasting (Fig. 2E). Serum glycerol levels remained unaltered in both WT and KO mice after sustained fasting in a time-dependent manner (Fig. 2F). To confirm lipolysis in adipose tissue, immunoblot analysis of lipolytic proteins, including hormone-sensitive lipase (HSL) and adipose triglyceride lipase (ATGL) was performed. Phosphorylation of HSL and expression of ATGL were markedly increased in fasted KO mice, suggesting that lipolysis signaling in adipose tissue was over activated (Fig. 2G). To address the mechanistic evidence of how lipolysis occurs in KO mice, we reasoned that ROS might be a key player in inducing lipolysis, as previously shown [13]. Hence, the WAT of mice was homogenized and incubated with the dye 2′7’-dichlorofluorescein diacetate (DCFH-DA) to measure total ROS. As expected, ROS generation was significantly increased in both fed and fasted KO mice compared to WT mice. In addition, ROS level in fasted KO mice was significantly higher than in fed mice (Fig. 2H). Together, these data indicate that catalase deficiency during sustained fasting resulted in a marked reduction in fat mass, which is mediated through lipolysis of WAT caused by ROS generation.

### Catalase deficiency during sustained fasting induces brown adipose tissue thermogenesis

**Fig. 3:**
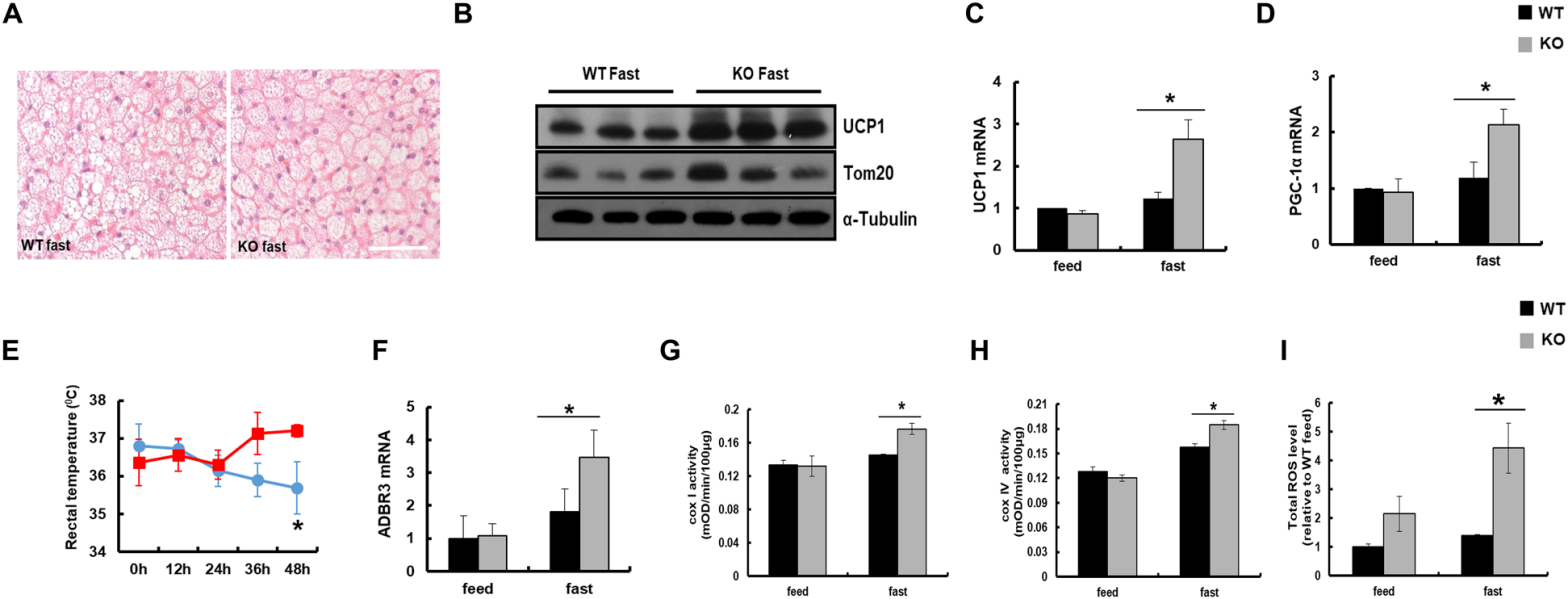
Catalase deficiency during sustained fasting increased BAT thermogenesis through mitochondrial activation. **A** Representative H&E staining of BAT from mice. Scale bar represents 20 μm. **B** Proteins were extracted from BAT of WT and KO mice and immunoblot analysis was performed using whole-cell lysates with the indicated antibodies (n = 3). α-Tubulin was used as a loading control. qPCR analysis of **C** UCP1 and **D** PGC-1α genes in BAT from mice. **E** Rectal body temperature was measured from mice fasted for indicated time (n = 6). **F** qPCR analysis of ADBR3. **G**, **H** Activities of mitochondrial complexes I and IV from BAT of mice. Details are described in Materials and Methods. **I** Total ROS was measured from BAT. Values represent mean ± SD (n = 3, 4). *P < 0.05 WT 48 h fasting versus KO 48 h of fasting

Lipolysis has been shown to provide a source of energy for brown fat thermogenesis [34, 35]. In addition, reduction of fat mass is accompanied by increased energy expenditure and intrascapular BAT (iBAT) thermogenesis [36]. As FFAs released from WAT were neither consumed nor accumulated in the liver in KO mice, we assumed these FFAs might be consumed by BAT for thermogenesis. To test BAT activation, BAT isolated from WT and KO mice was isolated and weighed. However we did not see any change in weight of BAT from experimental mice (Additional file 1: Fig.S5).Although, the morphological analysis of BAT by H&E staining showed with multilocular lipid droplets with slight reduced lipid deposition in KO mice after sustained fasting (Fig. 3A). It is well known that BAT activates UCP1 in the mitochondrial membrane to release potential energy as heat [34]. Expression of uncoupling proteins, UCP1 were markedly increased in BAT from KO mice after sustained fasting compared to WT mice (Fig. 3B). Furthermore, expression of the mitochondrial marker Tom20 was increased only in fasted KO mice. Consistently, mRNA levels of *UCP1* and *PGC-1α* were increased in fasted KO mice compared to WT mice (Fig. 3C, D). As thermogenesis protein was increased in KO mice, we measured the rectal temperature of mice. The rectal temperature was significantly higher in fasted KO mice than in WT mice after 24 h of fasting (Fig. 3E). It is well known that activation of BAT is regulated by the sympathetic nervous system via β3-adrenergic receptors (ADBR3) [37]. Q-PCR for *ADBR3* mRNA from BAT was performed, which revealed that expression of *ADBR3* was significantly increased in fasted KO mice compared to WT mice (Fig. 3F). To evaluate whether increased expression of mitochondrial proteins in fasted KO mice was associated with mitochondrial activity in BAT, the enzymatic activities of mitochondrial complex I (COX I) and complex IV (COX IV) were measured. The enzymatic activity of COX I and IV was significantly increased in BAT from KO mice after sustained fasting (Fig. 3G, H). ROS may also be the key player in inducing BAT thermogenesis as it induces lipolysis in WAT (Fig. 2H). To prove this, BAT was homogenized and incubated with DCFH-DA, and total ROS was measured. ROS generation was significantly increased in BAT of KO mice compared to WT mice on sustained fasting (Fig. 3I). These data suggest that FFAs released from excessive lipolysis of WAT may be consumed by the ROS-mediated thermogenic pathway of BAT in fasted KO mice.

### Catalase deficiency during sustained fasting facilitates the shuttling of FFA to the BAT but not in the liver through FA transporter

Fasting enhances lipid catabolism by hydrolyzing TG stored in adipocytes, which augments FFAs and glycerol in the liver to supply lipids to other tissues. We found that catalase deficiency during sustained fasting diminished hepatic lipid accumulation and augmented lipolysis to fuel the energy demand. Although lipolysis was induced, the liver did not obtain sufficient lipids during sustained fasting in KO mice. Thus, we hypothesized that the release of FFAs from fat tissues during sustained fasting might be shuttled to BAT rather than the liver in KO mice.

**Fig. 4:**
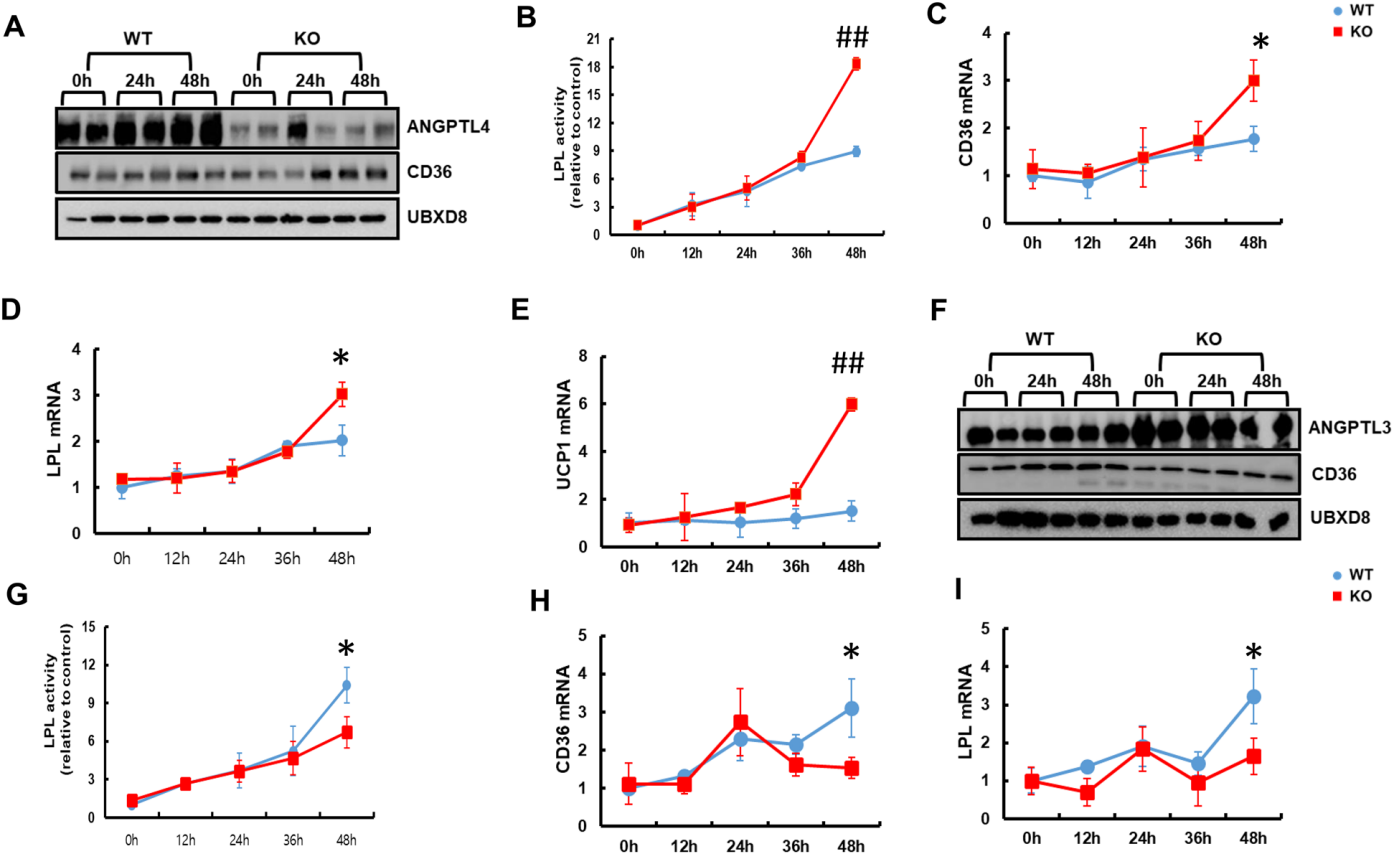
Catalase deficiency during sustained fasting facilitates the shuttling of free fatty acid to BAT through fatty acid receptor enzymes. **A** Proteins were extracted from BAT of mice fasted at indicated time and immunoblot analysis was performed using whole-cell lysates with indicated antibody (n = 2). **B** Isolated BAT from fasted mice at indicated time were homogenized and LPL activity was measured. qPCR analysis of **C** CD36, **D** LPL and **E** UCP1 in the BAT of mice fasted for indicated time. **F** Proteins were extracted from liver of mice fasted at indicated time and immunoblot analysis was performed using whole cell lysates with indicated antibodies (n = 2). **G** Isolated liver from fasted mice at indicated time were homogenized and LPL activity was measured. qPCR analysis of **H** CD36 and **I** LPL genes in liver of mice fasted for indicated time. Values represent mean ± SD (n = 3, 4). *P < 0.05 WT 48 h fasting versus KO 48 h of fasting; ^##^P < 0.001 WT 48 h fasting versus KO 48 h of fasting

During fasting, triglyceride-rich lipoprotein (TRL) transports lipids to the bloodstream, where lipids are catabolized by the action of LPL. Hence, through LPL and transmembrane receptor cluster of differentiation 36 (CD36), other tissues such as liver, muscle, and brown adipocytes get energy during nutrient deprivation [38, 39]. Recently, it was reported that during lipolysis, FFAs are delivered by TRL to the FA receptor enzyme CD36 and catabolized by LPL,that promotes sufficient energy needed for BAT activation [39]. Hence, BAT utilizes FA that are liberated from lipolysis as a source that activates UCP1 for thermogenesis [40, 41]. BAT activation inhibits ANGPTL4, an important regulator of LPL-mediated FA uptake into BAT. Shuttling of ANGPTL4 enhances LPL activity to uptake FA derived from circulating TRL to BAT activation [11]. Hence to test the activity of the FA receptor, immunoblot analysis was performed to check FA shuttling protein in BAT of mice fasted for 48 h. The protein level of ANGPTL4 was markedly decreased whereas CD36 was markedly increased in the BAT of KO mice during sustained fasting (Fig. 4A). On the contrary, LPL activity significantly increased after 36 h of fasting in the BAT of KO mice compared to that of WT mice (Fig. 4B). Consistent with LPL activity, mRNA levels of CD36 and LPL were also significantly increased in the BAT of KO mice compared to WT mice (Fig. 4C, D). FA taken up by CD36 and catabolized by LPL in BAT could be a major fuel source to activate UCP1 in the mitochondrial membrane. Herein, we observed that the mRNA level of UCP1 continuously increased after 12 h of sustained fasting in KO mice compared to WT mice (Fig. 4E). In contrast, immunoblot analysis with ANGPTL3 protein in the liver showed a marked increase whereas CD36 level was slightly decreased in expression up to 48 h during sustained fasting in KO mice compared to WT mice (Fig. 4F). The lipolytic activity of LPL decreased significantly during sustained fasting in the liver of KO mice (Fig. 4G). Furthermore, mRNA levels of LPL and CD36 were significantly decreased in KO mice during sustained fasting compared to WT mice (Fig. 4H, I). Together, these data indicate that catalase deficiency during sustained fasting facilitates the shuttling of FFAs to BAT through the FA receptor, CD36, and were catabolized by LPL.

### Both ROS generation and lipolysis by isoproterenol is attenuated by N-acetylcysteine in catalase deficient adipocytes

**Fig. 5:**
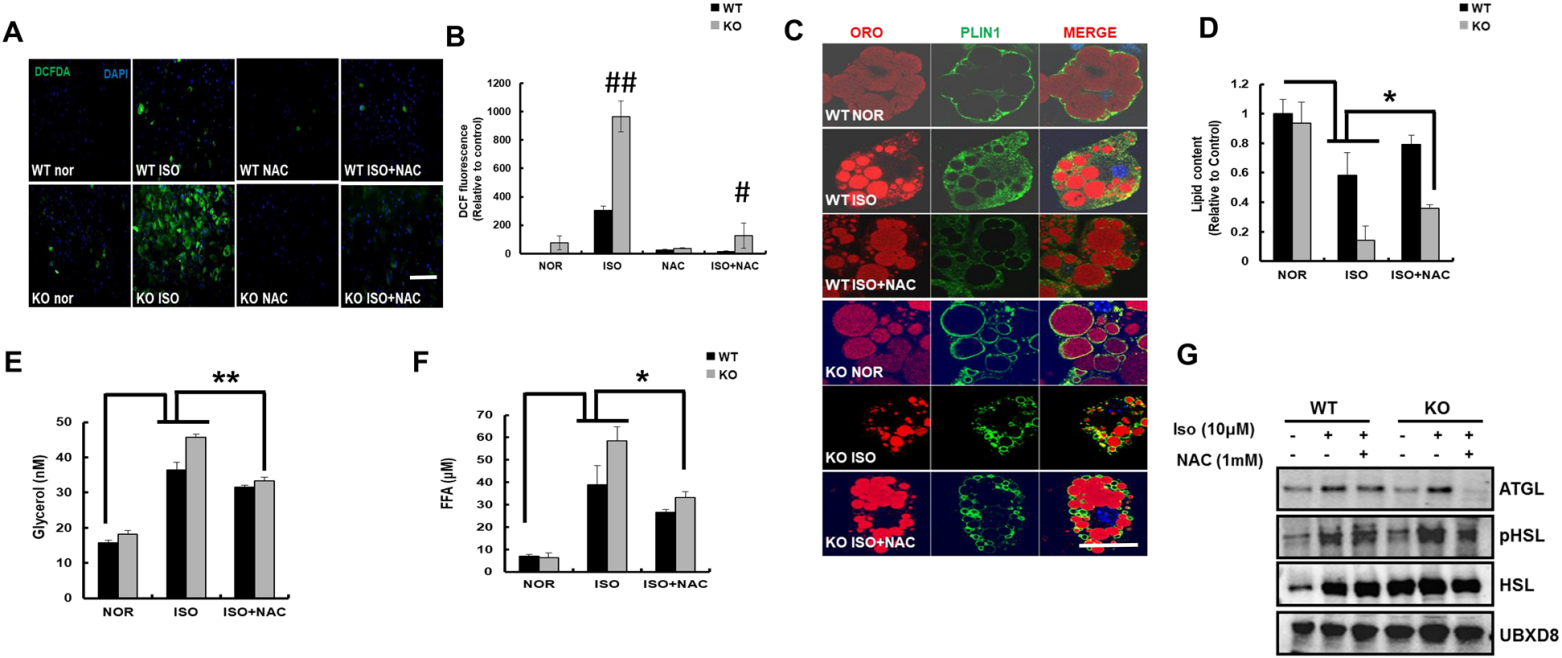
ROS generation induced lipolysis was suppressed by N-Acetylcysteine in catalase deficient adipocytes. **A** Representative fluorescence images of primary white adipocytes treated as indicated and fluorescence were measured with DCFH-DA and examined under a fluorescence microscope. Scale bar represents 100 μm. **B** Percentage of cells showing green fluorescence (corresponding to DCFH-DA) and fluorescence intensity of adipocytes. ^##^P < 0.001 WT ISO vs KO ISO; ^#^P < 0.001 KO ISO vs KO ISO + NAC. **C** Representative fluorescence images primary white adipocytes, fixed and immunostained with anti-PLIN1 (green), ORO (red), and DAPI (blue). Scale bar represents 5 μm. **D** The quantification of ORO positive cells in white adipocytes from C. *P < 0.05 WT nor vs WT ISO; WT ISO vs KO ISO; KO ISO vs KO ISO + NAC. **E** Serum glycerol; **P < 0.01 WT nor vs WT ISO; WT ISO vs KO ISO; KO ISO vs KO ISO + NAC and **F** FFA level in primary white adipocytes. *P < 0.05 WT nor vs WT ISO; WT ISO vs KO ISO; KO ISO vs KO ISO + NAC. **G** Proteins were extracted from treated adipocytes and immunoblot analysis was performed using whole-cell lysates with the indicated antibodies. Data represent mean ± SD of three independent experiments

To confirm that the induction of lipolysis strictly occurred from WAT and not due to any other reason including hormonal changes in vivo, we analyzed the autonomous induction of lipolysis from primary inguinal white adipose tissue (iWAT). To mimic the effects of fasting and lipolysis in vitro, isoproterenol was added to differentiated iWAT of WT and KO mice. Isoproterenol treatment significantly increased the green fluorescence signals derived from DCFH-DA staining, reflecting increased ROS generation only in KO mice (Fig. 5A, B). The fluorescent signal in isoproterenol-treated iWAT of KO mice was inhibited after the addition of N-acetylcysteine (NAC), confirming the effect of NAC on ROS generation. We were further interested in the role of ROS in isoproterenol-induced lipolysis. As expected, treatment of differentiated iWAT with isoproterenol resulted in lipolysis, observed as fragmented smaller lipid droplets and decreased fluorescent intensity of PLIN1 compared to WT (Fig. 5C). However, features of lipolysis were more obvious in differentiated iWAT from KO mice than those from WT mice (Fig. 5C, D). We also confirmed that treatment with NAC partially recovered lipolytic features, including the fluorescence intensity of PLIN1 and lipid content, of differentiated iWAT from WT and KO mice (Fig. 5C, D). In addition, the hydrolysis of cellular lipids induced by isoproterenol in differentiated iWAT of KO mice was more severe than that in WT mice, which was significantly inhibited by the addition of NAC. To determine whether NAC mitigated lipolysis in iWAT, we measured the levels of glycerol and FFAs (Fig. 5E, F). These levels significantly increased on treatment with isoproterenol, which was significantly attenuated by NAC in differentiated iWAT of both WT and KO mice. Consistently, the expression of lipolytic proteins, including phospho-HSL and ATGL, decreased on addition of NAC to catalase-KO adipocytes treated with isoproterenol (Fig. 5G). Taken together, these data suggest that the antioxidant NAC prevents lipolysis induced by ROS in catalase-KO primary white adipocytes.

### UCP1 activation in brown adipocytes induced by ROS is suppressed by N-acetylcysteine in catalase deficient adipocytes

**Fig. 6:**
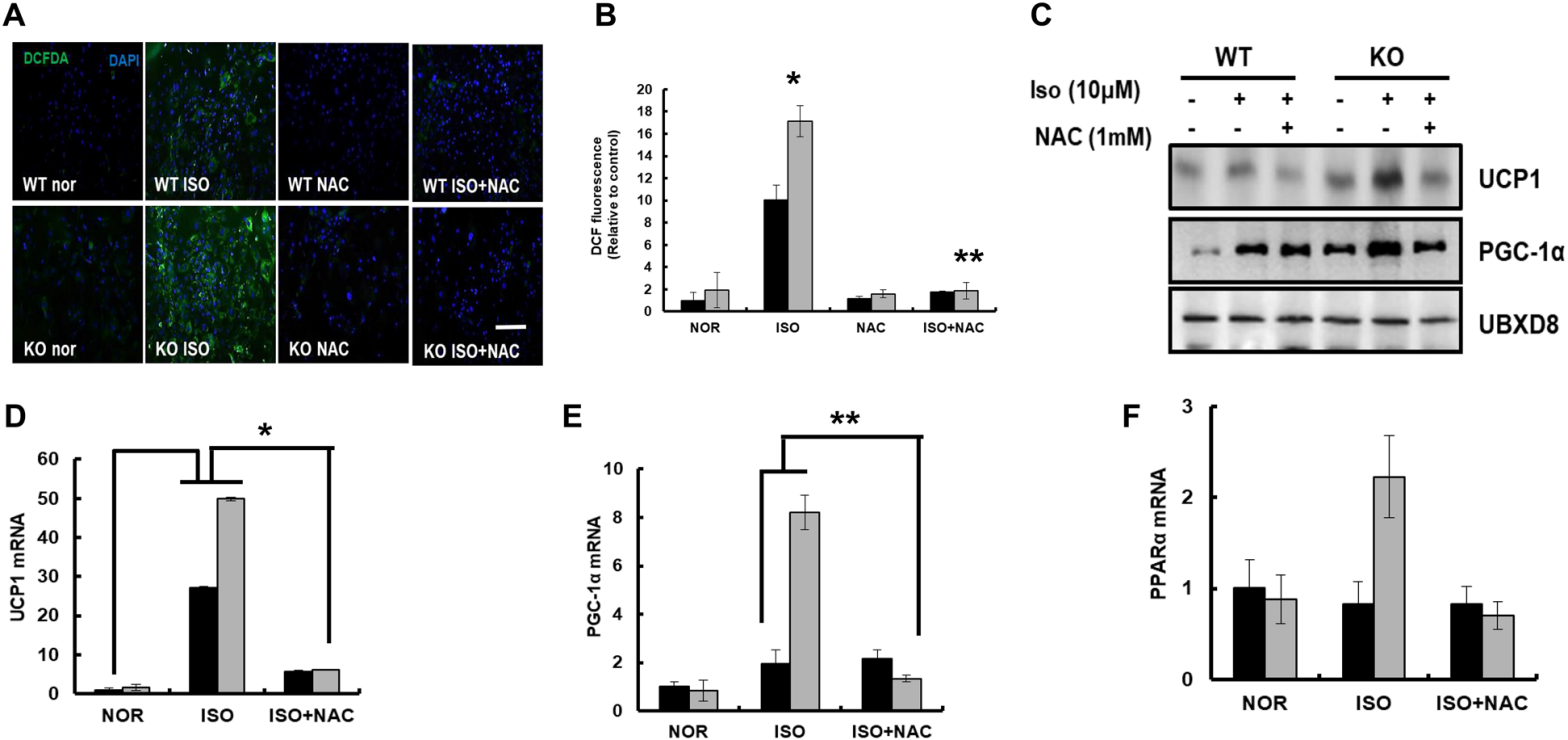
UCP1 activation in brown adipocytes induced by ROS was suppressed by N-Acetylcysteine in catalase deficient adipocytes. **A** Representative fluorescence images primary brown adipocytes treated as indicated and fluorescence were measured with DCFH-DA and examined under a fluorescence microscope. Scale bar represents 100 μm. **B** Percentage of cells showing green fluorescence (corresponding to DCFH-DA) and fluorescence intensity of adipocytes. *P < 0.05 WT ISO vs KO ISO; **P < 0.001 KO ISO vs KO ISO + NAC **C** Proteins were extracted from treated adipocytes and immunoblot analysis was performed using whole cell lysates with the indicated antibodies. qPCR analysis of **D** UCP1: *P < 0.01 WT nor vs WT ISO; WT ISO vs KO ISO; KO ISO vs KO ISO + NAC. **E** PGC-1α: **P < 0.05 WT ISO vs KO ISO; KO ISO vs KO ISO + NAC and **F** PPARα in isolated brown adipocytes. Data represent mean ± SD of three independent experiments

To assess the effect of ROS generated during fasting on BAT thermogenesis, differentiated BAT was treated with isoproterenol, and ROS generation was measured by DCFH-DA staining in the presence or absence of NAC (Fig. 6A). The results showed that treatment with isoproterenol increased the green fluorescence signals derived from DCFH-DA staining, reflecting an increase in ROS generation from differentiated BAT of WT and KO mice (Fig. 6A, B). However, there was a marked increase in the green fluorescence signals of DCFH-DA in differentiated BAT of KO mice compared to that of WT mice, which was inhibited by the addition of NAC. Next, proteins involved in uncoupling and mitochondrial enzymes were analyzed by immunoblot analysis and Q-PCR. The band intensities of, UCP1, and PGC-1α were dominant in isoproterenol-treated differentiated BAT from KO mice compared to those of WT mice (Fig. 6C). As expected, co-treatment with NAC diminished the expression of UCP1, and PGC-1α in isoproterenol-treated differentiated BAT of both WT and KO mice. The mRNA expression of *UCP1* was also increased in isoproterenol-treated differentiated BAT of both WT and KO mice, which was also diminished by the addition of NAC (Fig. 6D). Next, we measured the transcriptional activation of genes involved in mitochondrial biogenesis and β-oxidation, including *PGC1a* and *PPARα*, in differentiated BAT treated with isoproterenol (Fig. 6E, F). Treatment with isoproterenol predominantly increased the mRNA expression of *PGC-1α* and *PPARα* in differentiated BAT of KO mice, which was also clearly diminished by NAC. Together, these data suggest that treatment with fasting mimic isoproterenol results in ROS generation, which further activates the signaling pathway of WAT lipolysis and uncoupling events of BAT in KO mice. Furthermore, antioxidant NAC successfully prevented ROS-mediated lipolysis of WAT and uncoupling of BAT under fasting mimic conditions.

## Discussion

During fasting, TG in adipose tissue is hydrolyzed to FFAs and transferred to the liver, where they are utilized for energy production and stored in lipid droplets. In contrast, we found that hepatic TG accumulation was reduced in fasted KO mice. We speculated that lipolysis in fasted KO mice induces enhancement of β-oxidation, which promotes H_2_O_2_ generating oxidase in peroxisomes. The H_2_O_2_ generated may cause a decrease in lipid accumulation in hepatocytes. Our speculation is supported by a previous report that ROS is generated in catalase-deficient mice and in nutrient deprived cells [21, 22]. Additionally, the cellular redox state affects the activity of several enzymes involved in lipid metabolism in the liver [42].

Lipolysis induces the release of FFAs from white adipocytes and triggers BAT activation via UCP1 activation for thermogenesis [39–41]. To generate heat, BAT uses a large amount of TG to perform efficient mechanisms for continuous replacement of intracellular lipid storage. We showed that catalase deficiency during sustained fasting induces BAT thermogenesis and hence requires a surplus amount of lipid to fuel thermogenesis. Therefore, the fatty acid receptor enzyme CD36 is active in BAT, and continuously takes up the FA delivered by circulating TRL and catabolized by LPL [11, 43]. Taken together, we believe that FFAs released from white adipocytes are taken up by BAT through the activation of CD36. Meanwhile, activation of ANGPTL3, a negative regulator of LPL in hepatocytes, reduced fatty acid receptor enzymes, resulting in the liver not getting sufficient FA delivered by circulating TRL. This observation caused a decrease in hepatic lipid accumulation during sustained fasting in KO mice. Recently, the fatty acid receptor enzyme CD36 was shown to be involved in the degradation of lipids in the liver [44], which overlaps with our data that a decrease in fatty acid receptor enzymes lessened lipid accumulation in the liver. Next, we investigated how ANGPTL3 in the liver and ANGPTL4 in BAT regulate fatty acid receptor enzymes in circulating TRL during fasting. Although the exact mechanism remains to be elucidated, we hypothesize that ROS generation in KO mice might regulate ANGPTLs in the bloodstream. Our assumption is consistent with a previous report that ROS inhibits ANGPTL4 expression in adipocytes during hyperoxia [45, 46]. Hence, ROS generation in BAT might be a key player in the inhibition of ANGPTL4, which activates LPL to trigger BAT thermogenesis. Two members of the angiopoietin-like family, ANGPTL3 and ANGPTL4, modulate lipoprotein metabolism with differences in key mechanisms. In addition to tissue-specific localization, ANGPTL3 in the liver and ANGPTL4 in adipocytes, their protein structure and mode of inhibition of LPL activity are also different [3, 47]. Furthermore, we also showed that fat mass was significantly decreased in fasted KO mice, which might be due to enhanced lipolysis. Reduced fat mass is also accompanied by the activation of BAT thermogenesis [36]. Indeed, our data suggest that lipolysis is activated in fasted KO mice and that the FFAs released from WAT were unavailable for delivery to the liver for TG synthesis because BAT consumed them for thermogenesis.

As ROS increases in all the metabolic tissues including liver, WAT and BAT, we were keen to check the expression level of catalase and other antioxidant enzyme such as GPX and PRX3 at different time interval during fasting. We hypothesize that catalase expression might be also increased in WAT and BAT during sustained as in liver (Additional file 1: Fig. S1A–C). Hence, mice were fasted for different time intervals (0, 6, 12, 24, 36, and 48 h) and immunoblot analysis was performed using WAT and BAT homogenates to examine the differential expression of catalase, GPX and PRX3 (Additional file 1: Fig S6A, B). Although catalase expression was slightly increased in a time dependent manner after fasting in white adipocytes (Additional file 1: Fig.S6A), we did not observe any distinctive change of catalase in BAT and other antioxidant in both WAT and BAT (Additional file 1: Fig. S6A, B). To make it more clear we measured catalase activity in white and brown adipocytes from the mice fasted for 0, 24, 36 and 48 h (Additional file 1: Fig. S6C, D). The enzymatic activity of catalase was significantly increased during sustained fasting in both white and brown adipocytes. Further, we analysed the protein level of GPX and PRX III in WT and catalase KO mice in the liver, WAT, and BAT during sustained fasting. The expression level of both antioxidants was unaltered in the liver, WAT, and BAT, respectively (Additional file 1: Fig. S6 E–G). Hence we conclude that catalase might be a key player in metabolic tissues to regulates ROS during sustained fasting.

It is well known that UCP1 is responsible for non-shivering thermogenesis in BAT. However, the factor responsible for the production of heat by UCP1 in BAT during fasting is unclear. Many studies in mice and humans have shown that cold stimulation induces non-shivering thermogenesis in BAT. Moreover, liberation of FA from WAT by lipolysis is responsible for BAT thermogenesis during fasting [35, 40, 41]. As discussed above, FA released by lipolysis can be taken up by BAT for thermogenesis. Hence, fasting could also be a possible reason for BAT activation [41]. Other reports also suggest that BAT thermogenesis is activated by fasting, such as intermittent or every other day fasting, besides cold stress, to alleviate obesity [48, 49]. We also showed that ROS generation is responsible for lipolysis and BAT thermogenesis, concurrent with previous reports suggesting that BAT activation is associated with ROS generation [13–16]. Consistently, our study suggests that enhanced ROS generation induces UCP1 for thermogenesis in catalase-KO brown adipocytes.

Peroxisomes are abundant and play a vital role in the adipose tissue [50–53]. In adipocytes, they are closely associated with lipid droplets and play a key role during the differentiation of pre-adipocytes to mature adipocytes, suggesting that de novo peroxisome formation and transcriptional regulation of thermogenesis are interrelated [54]. We showed that the peroxisome number is decreased in the liver [22], whereas it is increased in BAT in KO mice during sustained fasting (Additional file 1: Fig. S7). We argued that the drastic appearance of peroxisomes might be specific to the tissue. In the liver due to sustained fasting, we can speculate that excessive ROS is induced for meeting energy demands, which might increase peroxisome oxidases to induce autophagic degradation of peroxisomes as a self-defense mechanism in catalase-deficient mice. In contrast, peroxisomes are critical for BAT thermogenesis, as reported earlier [54].

Collectively, our study proposes that controlling catalase activity would be a valuable approach for maintaining homeostatic balance in lipid metabolism.

## Materials and methods

### Animal treatments

Homozygous KO mice were kindly provided by Dr. Ye-Shih Ho (Institute of Environmental Health Sciences and Department of Biochemistry and Molecular Biology, Wayne State University, USA). Mice were interbred and experiment were designed as described previously [22]. Mice were maintained in accordance with the standard protocol approved by the Animal Care and Use Committee at Gwangju Institute of Science and Technology, Korea.

### Isolation, culture and differentiation of primary adipocytes

Both iBAT, isolated from newborn to 7 days old mice, and iWAT, isolated from 7–8 weeks old mice were minced and subjected to collagenase digestion (2 mg of collagenase in 2 mL of isolation buffer containing 0.123 M NaCl, 5 mM KCl, 1.3 mM CaCl_2_, 5 mM glucose, 100 mM HEPES, and 4% BSA) for 1 h at 37 °C in a shaker. The cell suspension was filtered through a 100 μm filter. The filtrate was centrifuged at 600 × g for 5 min. Pellets were resuspended in 2 mL SVF growth medium (DMEM consisting of 25 mM glucose, 20% FBS, 20 mM Hepes, 100 units/mL penicillin/streptomycin) and seeded on 6-, 12-, and 24 well plates in a humidified atmosphere of 5% CO_2_. After 2 days post confluency (designated day 0), differentiation was induced by addition of differentiation media (DMEM containing 0.5 mM IBMX, 0.5 μM dexamethasone, 20 nM insulin) and incubated in a humidified atmosphere of 5% CO_2_. At day 2, the medium was replaced with maintenance medium (DMEM medium containing 20 nM insulin and 0.5 μM rosiglitazone). This medium was renewed every 48 h until the adipocytes fully differentiated.

### Lipolysis induction in adipocytes

After cell differentiation, the medium was replaced with lipolytic medium (DMEM medium containing 1 g/L glucose, 5% FBS, 2% BSA) and supplemented with or without 10 μM isoproterenol (I6504; Sigma-Aldrich) and incubated in a humidified atmosphere containing 5% CO_2_. The lipolytic experiments were carried out for 12 h. Cultures were frequently observed with a phase contrast microscope. After 12 h of incubation, culture medium from the cells was extracted. FFAs (Cayman #700310) and, glycerol (Biovision #K630-100) were measured from media of treated cell according to the manufacturer’s protocol.

### Measurement of hepatic TG levels

Serum TG levels were measured using a 7600 Clinical Analyzer (Hitachi). To measure hepatic TG, 50 mg of liver tissue was homogenized, and TG was measured using a Triglyceride Colorimetric Assay kit (Cayman, #10010303) according to the manufacturer’s protocol.

### Histological analysis

For histological analysis, tissues were fixed in 10% formalin solution, embedded in paraffin, and sectioned. H&E staining were performed as described previously [22].

For immunofluorescence (IF) staining of tissue and primary adipocytes, snap-frozen tissues were sectioned using a cryostat and then defrosted at room temperature for 1 h. The primary adipocytes and tissue sections were fixed in 4% paraformaldehyde (HT5014; Sigma-Aldrich) at room temperature for 20 min, washed thrice in PBS, incubated with 0.2% Triton X-100 for 5 min, and rinsed in PBS. The sections were blocked with 5% goat serum with 0.1% Triton X-100, whereas adipocytes were blocked with 3% bovine serum albumin (BSA) for 1 h at room temperature. Then, both were incubated with the target protein antibody (1:200) with their respective blocking solution overnight at 4 °C. The next day, the tissues and adipocytes were again washed thrice with PBS, stained with Alexa Fluor-conjugated secondary antibody in blocking solution at room temperature in the dark for 1 h, washed twice with PBS, and incubated with 10 μM DAPI in PBS at room temperature for 10 min. Images were acquired and analyzed using an Olympus FluoView 1000 confocal laser scanning system. The slides were scored in a blinded manner.

### Oil Red O (ORO) staining

Cryosectioned liver samples and primary adipocytes were fixed with paraformaldehyde for 20 min and washed twice with distilled water. ORO solution (01391; Sigma-Aldrich) was added to the samples for 1 h at room temperature. The sections were washed with distilled water and counterstained with hematoxylin solution for 1 min whereas adipocytes were washed with PBS and incubated with 10 μM DAPI in PBS at room temperature for 10 min. Images were acquired and analyzed using a light microscope for tissue and fluorescence microscope for adipocytes. The slides were scored in a blinded manner.

### Western blot analysis

To determine the expression levels of target protein, western blot from whole cell analysis using tissues sample and primary adipocytes were performed as described previously [21, 22]. The antibodies used and their sources are listed in Additional file 1: Table S1.

### Measurement of total FAs

Liver tissue (~ 50 mg) from the experimental mice was homogenized using a TissueLyzer (Qiagen). Heneicosanoic acid as an internal standard was added to the samples, and FA were extracted by the Folch method [55]. For FA analysis, base hydrolysis using KOH was performed, followed by neutralization with HCl. Methyl esterification was performed with BCl3-MeOH at 60 °C for 30 min, and the FA content was analyzed by gas chromatography-mass spectrometry (GC–MS) analysis using 7890A/5975A (Agilent). All standards, including the internal standard for calibration, were purchased from Avanti Polar Lipids and Sigma-Aldrich.

### Quantitative reverse transcription-polymerase chain reaction (qPCR)

qPCR from tissues lysates and primary adipocytes were analysed as described earlier [22]. The primer sequences are listed in Additional file 1: Table S2.

### Mitochondrial complex activity

Liver and BAT were homogenized with buffer provided in the Complex I Enzyme Activity Microplate Assay Kit (ab109721, Abcam Biotech, UK) and Complex IV Rodent Enzyme Activity Microplate Assay Kit (ab109911, Abcam Biotech). Mitochondrial complex I and IV activities were measured according to the manufacturer’s instructions.

### Determination of LPL activity

Liver and BAT tissues were homogenized in ice-cold PBS. The centrifuged supernatant was collected and the LPL activity was measured using the LPL activity assay kit (Biovision, # K72-100).

### Determination of ROS production

ROS production from tissue lysates and adipocytes were performed as previously described [21, 22].

### Terminal deoxynucleotidyl transferase dUTP nick-end (TUNEL) labeling assay

TUNEL assay from paraffin embedded WAT section were performed as described previously [22].

### Catalase activity

Liver, WAT, and BAT tissues were homogenized in ice-cold PBS. The sample were centrifuged at 4 °C at 10,000 × *g* and supernatant was collected and catalase activity was measured using the catalase activity assay kit (abcam, # ab83464).

### Statistical analysis

All values are presented as mean ± standard deviation (SD). One-way analysis of variance was used to compare means, and P-values < 0.05 were considered statistically significant.

## Supplementary Information

**Additional file 1: Fig. S1.** Catalase expression was specifically increased during sustained fasting. **Fig. S2.** The expression of catalase was successfully depleted in mice. **Fig. S3.** Sustained fasting decreased the level of free fatty acid in serum of catalase KO mice. **Fig. S4.** Catalase deficiency during sustained fasting did not induce inflammatory response, and lipodystrophy in adipocytes. **Fig. S5.** Catalase deficiency during sustained fasting did not show any morphological change in BAT. **Fig S6.** Catalase activity was significantly increased during sustained fasting with no change in other antioxidant enzyme. **Fig. S7.** Lipolysis by isoproterenol increased the peroxisomal enzyme in primary BAT of catalase KO mice. **Table S1.** List of antibodies used in this study. **Table S2.** List of primer sequence used for Q-PCR.

## Data Availability

All data generated and/or analyzed in this study are available from the corresponding author on reasonable request.

## Abbreviations

ANGPTLs: Angiopoietin-like proteins
BAT: Brown Adipose Tissue
CD36: Cluster of differentiation 36
FA: Fatty Acids
FAO: Fatty Acid Oxidation
FFAs: Free Fatty Acids
KO: Knock Out
LPL: Lipoprotein Lipase
ROS: Reactive Oxygen Species
TG: Triglyceride
UCP1: Uncoupling protein 1
VLCFA: Very Long Chain Fatty Acid
WAT: White Adipose Tissue
WT: Wild Type

## References

[1] Lee YK, Sohn JH, Han JS, Park YJ, Jeon YG, Ji Y, Dalen KT, Sztalryd C, Kimmel AR, Kim JB (2018). Perilipin 3 deficiency stimulates thermogenic beige adipocytes through PPARα activation. Diabetes.

[2] Heeren J, Scheja L (2018). Brown adipose tissue and lipid metabolism. Curr Opin Lipidol.

[3] Shan L, Yu XC, Liu Z, Hu Y, Sturgis LT, Miranda ML, Liu Q (2009). The angiopoietin-like proteins ANGPTL3 and ANGPTL4 inhibit lipoprotein lipase activity through distinct mechanisms. J Biol Chem.

[4] Finn PF, Dice JF (2006). Proteolytic and lipolytic responses to starvation. Nutrition.

[5] Lee JN, Dutta RK, Kim SG, Lim JY, Kim SJ, Choe SK, Yoo KW, Song SR, Park DS, So HS (2013). Fenofibrate, a peroxisome proliferator-activated receptor α ligand, prevents abnormal liver function induced by a fasting-refeeding process. Biochem Biophys Res Commun.

[6] Wang Y, McNutt MC, Banfi S, Levin MG, Holland WL, Gusarova V, Gromada J, Cohen JC, Hobbs HH (2015). Hepatic ANGPTL3 regulates adipose tissue energy homeostasis. Proc Natl Acad Sci U S A.

[7] Santulli G (2014). Angiopoietin-like proteins: a comprehensive look. Front Endocrinol (Lausanne).

[8] Quagliarini F, Wang Y, Kozlitina J, Grishin NV, Hyde R, Boerwinkle E, Valenzuela DM, Murphy AJ, Cohen JC, Hobbs HH (2012). Atypical angiopoietin-like protein that regulates ANGPTL3. Proc Natl Acad Sci U S A.

[9] Koishi R, Ando Y, Ono M, Shimamura M, Yasumo H, Fujiwara T, Horikoshi H, Furukawa H (2002). Angptl3 regulates lipid metabolism in mice. Nat Genet.

[10] Conklin D, Gilbertson D, Taft DW, Maurer MF, Whitmore TE, Smith DL, Walker KM, Chen LH, Wattler S, Nehls M, Lewis KB (1999). Identification of a mammalian angiopoietin-related protein expressed specifically in liver. Genomics.

[11] Dijk W, Heine M, Vergnes L, Boon MR, Schaart G, Hesselink MK, Reue K, van Marken Lichtenbelt WD, Olivecrona G, Rensen PC, Heeren J, Kersten S (2015). ANGPTL4 mediates shuttling of lipid fuel to brown adipose tissue during sustained cold exposure. Elife.

[12] Kersten S, Mandard S, Tan NS, Escher P, Metzger D, Chambon P, Gonzalez FJ, Desvergne B, Wahli W (2000). Characterization of the fasting-induced adipose factor FIAF, a novel peroxisome proliferator-activated receptor target gene. J Biol Chem.

[13] Krawczyk SA, Haller JF, Ferrante T, Zoeller RA, Corkey BE (2012). Reactive oxygen species facilitate translocation of hormone sensitive lipase to the lipid droplet during lipolysis in human differentiated adipocytes. PLoS ONE.

[14] Chouchani ET, Kazak L, Jedrychowski MP, Lu GZ, Erickson BK, Szpyt J, Pierce KA, Laznik-Bogoslavski D, Vetrivelan R, Clish CB, Robinson AJ, Gygi SP, Spiegelman BM (2016). Mitochondrial ROS regulate thermogenic energy expenditure and sulfenylation of UCP.1. Nature.

[15] Shabalina IG, Vrbacký M, Pecinová A, Kalinovich AV, Drahota Z, Houštěk J, Mráček T, Cannon B, Nedergaard J (2014). ROS production in brown adipose tissue mitochondria: the question of UCP1-dependence. Biochim Biophys Acta.

[16] Dlasková A, Clarke KJ, Porter RK (2010). The role of UCP 1 in production of reactive oxygen species by mitochondria isolated from brown adipose tissue. Biochim Biophys Acta.

[17] Phaniendra A, Jestadi DB, Periyasamy L (2015). Free radicals: properties, sources, targets, and their implication in various diseases. Indian J Clin Biochem.

[18] Schrader M, Fahimi HD (2006). Peroxisomes and oxidative stress. Biochim Biophys Acta.

[19] Ho YS, Xiong Y, Ma W, Spector A, Ho DS (2004). Mice lacking catalase develop normally but show differential sensitivity to oxidant tissue injury. J Biol Chem.

[20] Takahara S, Miyamoto H (1948). Three cases of progressive oral gangrene due to lack of catalase in the blood. Nippon Jibi-Inkoka Gakkai Kaiho.

[21] Lee JN, Dutta RK, Maharjan Y, Liu ZQ, Lim JY, Kim SJ, Cho DH, So HS, Choe SK, Park R (2018). Catalase inhibition induces pexophagy through ROS accumulation. Biochem Biophys Res Commun.

[22] Dutta RK, Maharjan Y, Lee JN, Park C, Ho YS, Park R (2021). Catalase deficiency induces reactive oxygen species mediated pexophagy and cell death in the liver during prolonged fasting. BioFactors.

[23] Fransen M, Nordgren M, Wang B, Apanasets O (2012). Role of peroxisomes in ROS/RNS-metabolism: implications for human disease. Biochim Biophys Acta.

[24] Yao C, Behring JB, Shao D, Sverdlov AL, Whelan SA, Elezaby A, Yin X, Siwik DA, Seta F, Costello CE, Cohen RA, Matsui R, Colucci WS, McComb ME, Bachschmid MM (2015). Overexpression of catalase diminishes oxidative cysteine modifications of cardiac proteins. PLoS ONE.

[25] Paglialunga S, Ludzki A, Root-McCaig J, Holloway GP (2015). In adipose tissue, increased mitochondrial emission of reactive oxygen species is important for short-term high-fat diet-induced insulin resistance in mice. Diabetologia.

[26] Amos DL, Robinson T, Massie MB, Cook C, Hoffsted A, Crain C, Santanam N (2017). Catalase overexpression modulates metabolic parameters in a new 'stress-less' leptin-deficient mouse model. Biochim Biophys Acta Mol Basis Dis.

[27] Leone TC, Weinheimer CJ, Kelly DP (1999). A critical role for the peroxisome proliferator-activated receptor alpha (PPARalpha) in the cellular fasting response: the PPARalpha-null mouse as a model of fatty acid oxidation disorders. Proc Natl Acad Sci U S A.

[28] Rao MS, Reddy JK (2001). Peroxisomal beta-oxidation and steatohepatitis. Semin Liver Dis.

[29] Hashimoto T, Cook WS, Qi C, Yeldandi AV, Reddy JK, Rao MS (2000). Defect in peroxisome proliferator-activated receptor a-inducible fatty acid oxidation determines the severity of hepatic steatosis in response to fasting. J Biol Chem.

[30] Kersten S, Seydoux J, Peters JM, Gonzalez FJ, Desvergne B, Wahli W (1999). Peroxisome proliferator–activated receptor alpha mediates the adaptive response to fasting. J Clin Invest.

[31] Alves-Bezerra M, Cohen DE (2017). Triglyceride metabolism in the liver. Compr Physiol.

[32] Nielsen TS, Møller N (2014). Adipose triglyceride lipase and G0/G1 switch gene 2: approaching proof of concept. Diabetes.

[33] Liu L, Jiang Q, Wang X (2014). Adipose-specific knockout of SEIPIN/BSCL2 results in progressive lipodystrophy. Diabetes.

[34] Chondronikola M, Volpi E, Børsheim E, Porter C, Saraf MK, Annamalai P, Yfanti C, Chao T, Wong D, Shinoda K, Labbė SM, Hurren NM, Cesani F, Kajimura S, Sidossis LS (2016). Brown adipose tissue activation is linked to distinct systemic effects on lipid metabolism in humans. Cell Metab.

[35] Shin H, Ma Y, Chanturiya T, Cao Q, Wang Y, Kadegowda AKG, Jackson R, Rumore D, Xue B, Shi H, Gavrilova O, Yu L (2017). Lipolysis in brown adipocytes is not essential for cold-induced thermogenesis in mice. Cell Metab.

[36] Doan KV, Kinyua AW, Yang DJ, Ko CM, Moh SH, Shong KE, Kim H, Park SK, Kim DH, Kim I, Paik JH, DePinho RA, Yoon SG, Kim IY, Seong JK, Choi YH, Kim KW (2016). FoxO1 in dopaminergic neurons regulates energy homeostasis and targets tyrosine hydroxylase. Nat Commun.

[37] Szentirmai É, Kapás L (2017). The role of the brown adipose tissue in β3-adrenergic receptor activation-induced sleep, metabolic and feeding responses. Sci Rep.

[38] Young SG, Zechner R (2013). Biochemistry and pathophysiology of intravascular and intracellular lipolysis. Genes Dev.

[39] Townsend KL, Tseng YH (2014). Brown fat fuel utilization and thermogenesis. Trends Endocrinol Metab.

[40] Abumrad NA (2017). The liver as a hub in thermogenesis. Cell Metab.

[41] Cannon B, Nedergaard J (2017). What Ignites UCP1?. Cell Metab.

[42] Serviddio G, Bellanti F, Vendemiale G (2013). Free radical biology for medicine: learning from nonalcoholic fatty liver disease. Free Radic Biol Med.

[43] Bartelt A, Bruns OT, Reimer R, Hohenberg H, Ittrich H, Peldschus K, Kaul MG, Tromsdorf UI, Weller H, Waurisch C, Eychmüller A, Gordts PL, Rinninger F, Bruegelmann K, Freund B, Nielsen P, Merkel M, Heeren J (2011). Brown adipose tissue activity controls triglyceride clearance. Nat Med.

[44] Li Y, Yang P, Zhao L, Chen Y, Zhang X, Zeng S, Wei L, Varghese Z, Moorhead JF, Chen Y, Ruan XZ (2019). CD36 plays a negative role in the regulation of lipophagy in hepatocytes through an AMPK-dependent pathway. J Lipid Res.

[45] Quintero P, González-Muniesa P, García-Díaz DF, Martínez JA (2012). Effects of hyperoxia exposure on metabolic markers and gene expression in 3T3-L1 adipocytes. J Physiol Biochem.

[46] Quintero P, Gonzalez-Muniesa P, Martinez JA (2012). Influence of different oxygen supply on metabolic markers and gene response in murine adipocytes. J Biol Regul Homeost Agents.

[47] Biterova E, Esmaeeli M, Alanen HI, Saaranen M, Ruddock LW (2018). Structures of Angptl3 and Angptl4, modulators of triglyceride levels and coronary artery disease. Sci Rep.

[48] Kim KH, Kim YH, Son JE, Lee JH, Kim S, Choe MS, Moon JH, Zhong J, Fu K, Lenglin F, Yoo JA, Bilan PJ, Klip A, Nagy A, Kim JR, Park JG, Hussein SM, Doh KO, Hui CC, Sung HK (2017). Intermittent fasting promotes adipose thermogenesis and metabolic homeostasis via VEGF-mediated alternative activation of macrophage. Cell Res.

[49] Li G, Xie C, Lu S, Nichols RG, Tian Y, Li L, Patel D, Ma Y, Brocker CN, Yan T, Krausz KW, Xiang R, Gavrilova O, Patterson AD, Gonzalez FJ (2017). Intermittent fasting promotes white adipose browning and decreases obesity by shaping the gut microbiota. Cell Metab.

[50] Novikoff AB, Novikoff PM (1982). Microperoxisomes and peroxisomes in relation to lipid metabolism. Ann N Y Acad Sci.

[51] Novikoff AB, Novikoff PM, Rosen OM, Rubin CS (1980). Organelle relationships in cultured 3T3-L1 preadipocytes. J Cell Biol.

[52] Pavelka M, Goldenberg H, Hüttinger M, Kramar R (1976). Enzymic and morphological studies on catalase positive particles from brown fat of cold adapted rats. Histochemistry.

[53] Kong J, Ji Y, Jeon YG, Han JS, Han KH, Lee JH, Lee G, Jang H, Choe SS, Baes M, Kim JB (2020). Spatiotemporal contact between peroxisomes and lipid droplets regulates fasting-induced lipolysis via PEX5. Nat Commun.

[54] Park H, He A, Tan M, Johnson JM, Dean JM, Pietka TA, Chen Y, Zhang X, Hsu FF, Razani B, Funai K, Lodhi IJ (2019). Peroxisome-derived lipids regulate adipose thermogenesis by mediating cold-induced mitochondrial fission. J Clin Invest.

[55] Folch J, Lees M, Sloane Stanley GH (1957). A simple method for the isolation and purification of total lipides from animal tissues. J Biol Chem.

