# Supplementary material for: Catalase deficiency facilitates the shuttling of free fatty acid to brown adipose tissue through lipolysis mediated by ROS during sustained fasting
Source: Cell Biosci. 2021 Dec 7;11:201. doi: 10.1186/s13578-021-00710-5 (PMC8650429; doi:10.1186/s13578-021-00710-5)
Supplement: Supplementary file 1 — Additional file 1: Fig. S1. Catalase expression was specifically increased during sustained fasting. Fig. S2. The expression of catalase was successfully depleted in mice. Fig. S3. Sustained fasting decreased the level of free fatty acid in serum of catalase KO mice. Fig. S4. Catalase deficiency during sustained fasting did not induce inflammatory response, and lipodystrophy in adipocytes. Fig. S5. Catalase deficiency during sustained fasting did not show any morphological change in BAT. Fig S6. Catalase activity was significantly increased during sustained fasting with no change in other antioxidant enzyme. Fig. S7. Lipolysis by isoproterenol increased the peroxisomal enzyme in primary BAT of catalase KO mice. Table S1. List of antibodies used in this study. Table S2. List of primer sequence used for Q-PCR. [file 13578_2021_710_MOESM1_ESM.docx]

**Additional file 1
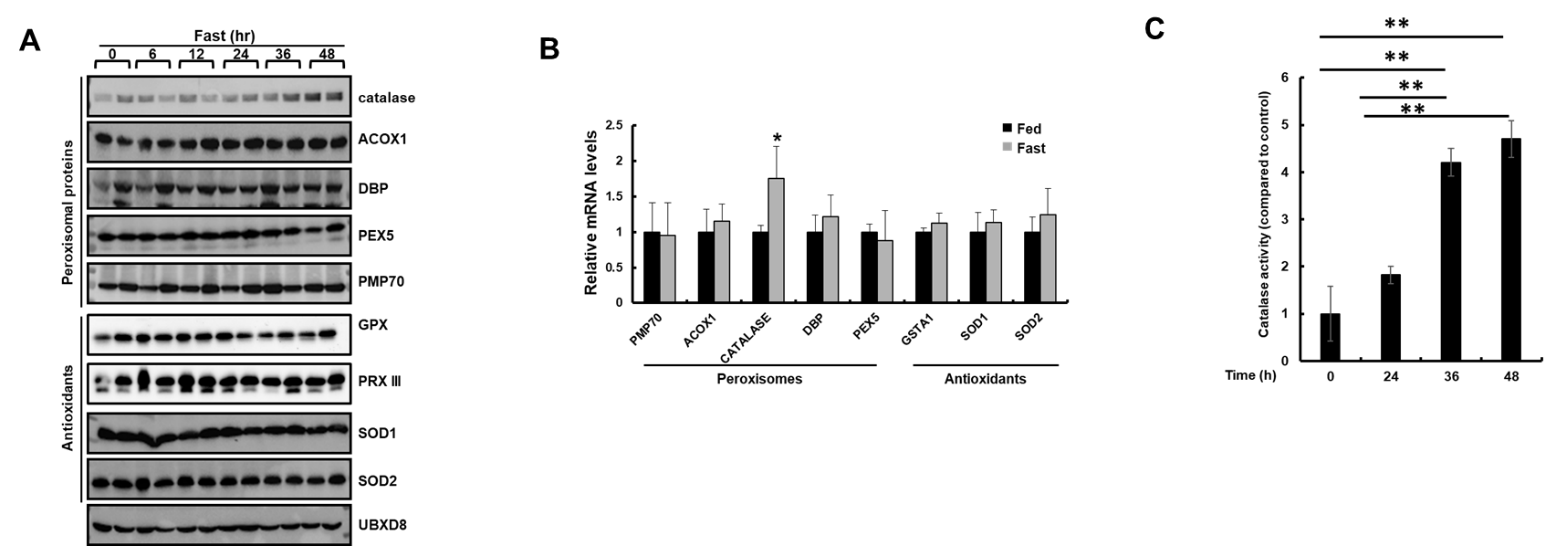
**

**Fig. S1. Catalase expression was specifically increased during sustained fasting**

(A) Liver samples from WT mice fasted at indicated time were homogenized and immunoblot analysis was performed. Protein expression was measured using whole-cell lysates with the indicated antibodies. (B) qPCR analysis of peroxisomes and antioxidant genes from the liver of WT mice fed and fasted for 48 h. Values represent mean ± SD (n = 3, 4). *P < 0.05 versus WT fed. (C) Catalase activity from liver of mice fasted for indicated time were measured. Values represent mean ± SD (n =4). **P < 0.001; *P < 0.05.

**
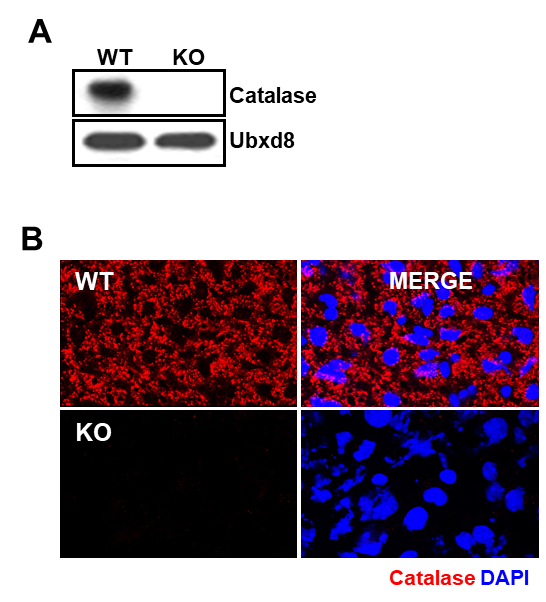
**

**Fig. S2. The expression of catalase was successfully depleted in mice**

(A) Liver samples from WT and catalase KO mice were homogenized and immunoblot analysis were performed from whole cell lysates with anti-catalase. (B) Representative fluorescence images of liver, fixed and immunostained with anti-catalase (red) and DAPI (blue).

**
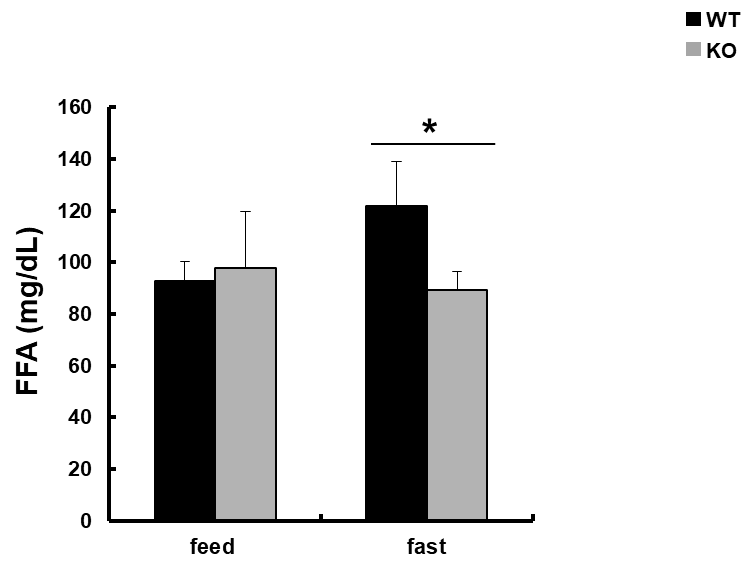
**

**Fig. S3. Sustained fasting decreased the level of free fatty acid in serum of catalase KO mice.**

FFA level was analyzed from serum of mice fasted for 0 and 48 h. *P <0.05 WT 48 h fasting versus KO 48 h of fasting

**
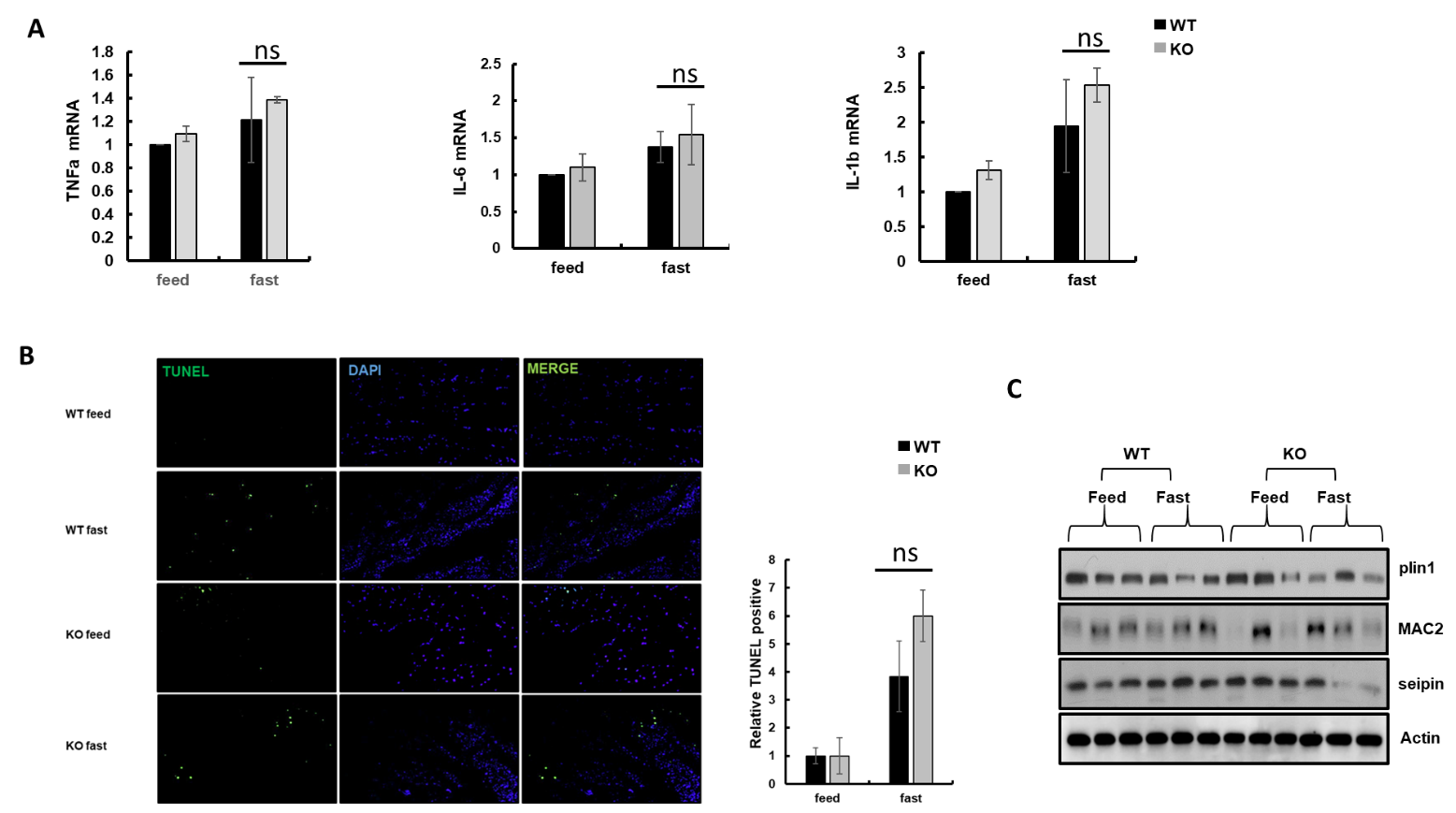
**

**Fig. S4. Catalase deficiency during sustained fasting did not induce inflammatory response, and lipodystrophy in adipocytes**

(A) qRT-PCR analysis for TNF-α, IL-6 and IL-1β was performed from WAT of experimental mice. (B) TUNEL assay of liver tissue prepared from mice as in A. TUNEL-positive nuclei were indicated in green. Quantitative TUNEL assay data. Values represent mean ± SD (n = 3); ns: non-significant. (C) Immunoblot analysis was done from whole-cell lysates with indicated antibodies.


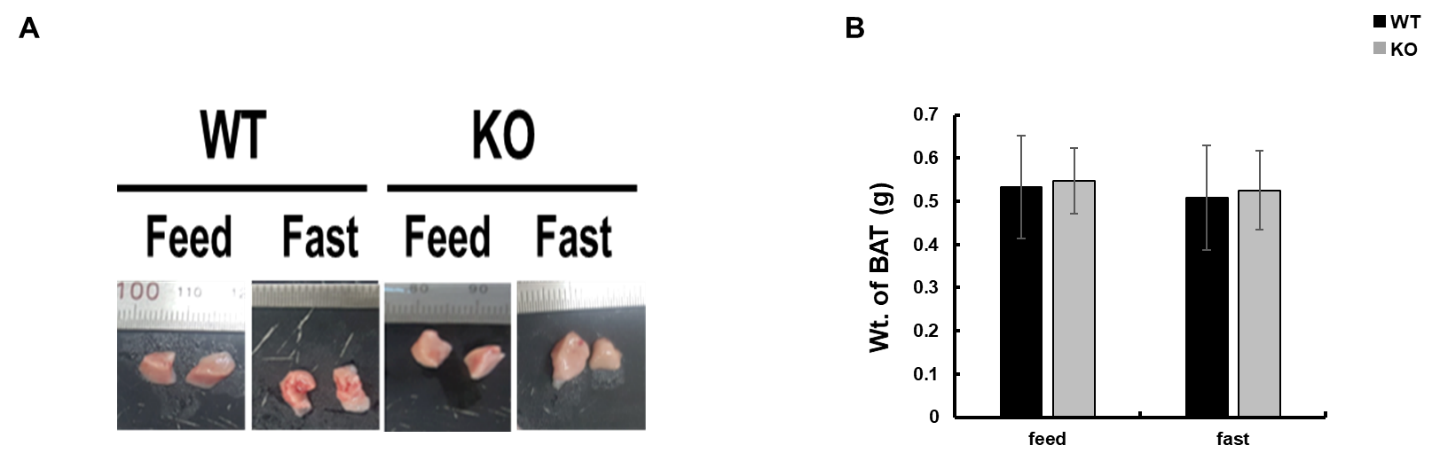


**Fig. S5. Catalase deficiency during sustained fasting did not show any morphological change in BAT**

(A) Images of BAT tissue from WT and catalase KO mice fed and or fasted for 48 h (n = 6). (B) Analytical weight of BAT from mice as in A in gram (g).

**
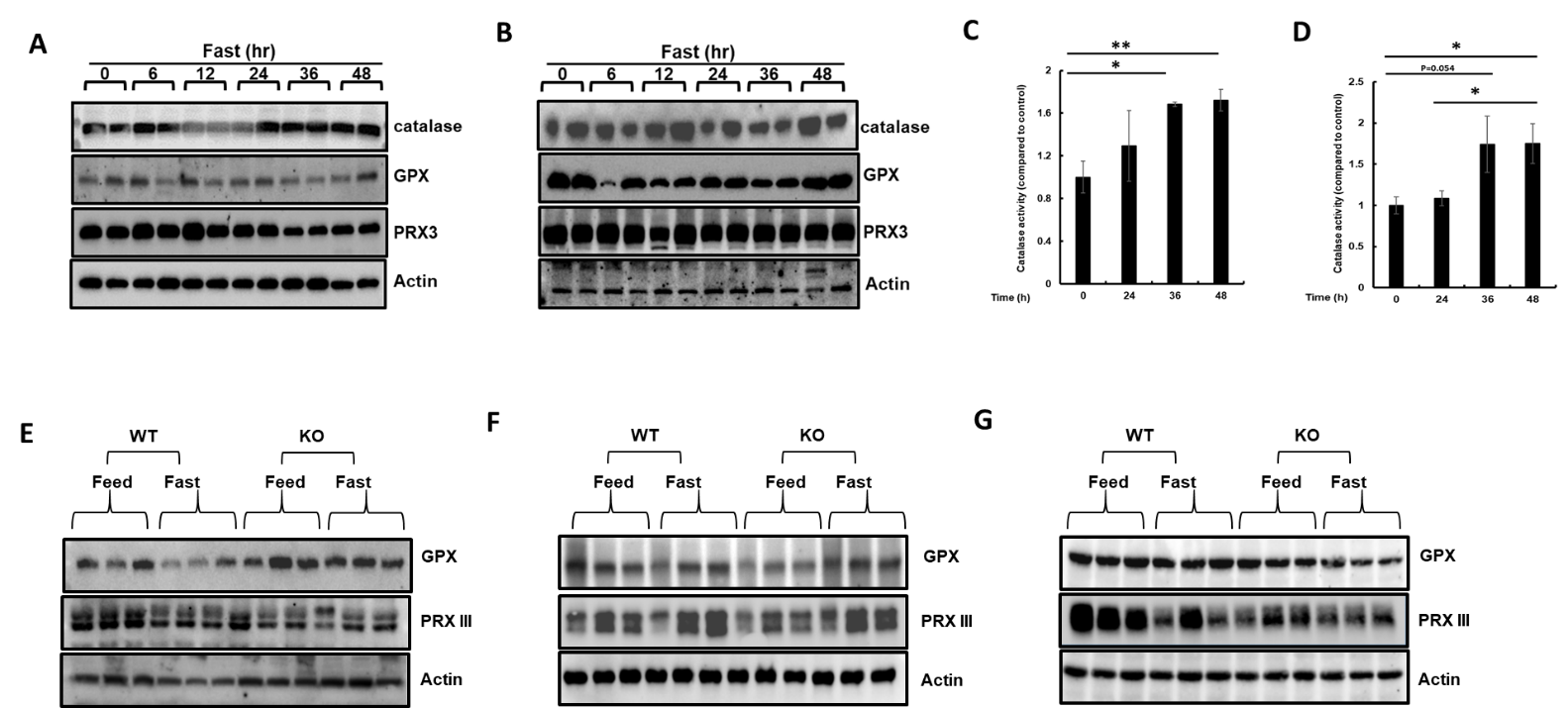
**

**Fig S6. Catalase activity was significantly increased during sustained fasting with no change in other antioxidant enzyme**

(A) WAT and (B) BAT from mice fasted at indicated time were homogenized and immunoblot analysis was performed for indicated antibodies. Anti-actin was used as a loading control. Catalase activity in (C) WAT and (D) BAT from mice fasted for indicated time intervals were measured. Values represent mean ± SD (n =4). **P < 0.001; *P < 0.01.

WT and catalase KO mice fed and or fasted for 48 h. Tissues sample from (E) liver , (F) WAT and (G) BAT were homogenized for immunoblot analysis with indicated antibodies.

**
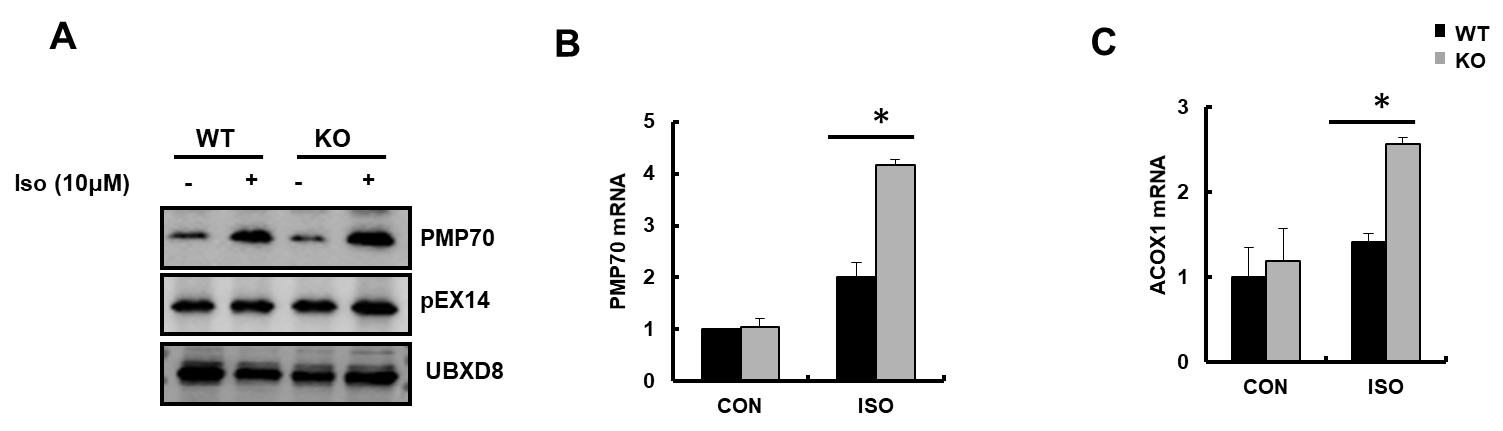
**

**Fig. S7. Lipolysis by isoproterenol increased the peroxisomal enzyme in primary BAT of catalase KO mice**

(A) Total cell lysates prepared from isoproterenol treated brown adipocytes were immunoblotted with antibodies indicated. Q-PCR analysis of (B) PMP70 and (C) ACOX1 in treated brown adipocytes. *P <0.05 WT ISO versus KO ISO.

**Table S1. List of antibodies used in this study**

| **antibody** | **Application** | **Dilution** | **company** | **cat. No.** | **source** |
| --- | --- | --- | --- | --- | --- |
| ACOX1 | WB | 1:1000 | proteintech | 10957-1-AP | rabbit |
| TOM20(F-10) | WB | 1:1000 | santa cruz | sc-17764 | Mouse |
| catalase | WB and IF | 1:1000 and 1:500 | abcam | ab4877 | rabbit |
| ADFP (Plin2) | WB | 1:1000 | abcam | ab52355 | rabbit |
| pex5 | WB | 1:1000 | genetex | GTX109798 | rabbit |
| PMP70 | WB and IF | 1:2000 and 1:500 | abcam | ab3421 | rabbit |
| ANGPTL3 | WB | 1:1000 | abcam | Ab84035 | rabbit |
| ANGPTL4 | WB | 1:1000 | Santa cruz | sc-373761 | mouse |
| CD36 | WB | 1:1000 | Novus biologicals | NB400-144 | rabbit |
| DBP | WB | 1:1000 | Origene | TA308904 | rabbit |
| UBXD8 | WB | 1:2000 |  |  | rabbit |
| Seipin | WB | 1:1000 | abnova | H00026580-A02 | Mouse |
| PGC-1α | WB | 1:1000 | santa cruz | sc-13067 | rabbit |
| SOD1 | WB | 1:1000 | Santa cruz | sc-11407 | rabbit |
| SOD2 | WB | 1:1000 | Santa cruz | sc-30080 | rabbit |
| PLIN1 | WB and IF | 1:1000 and 1:500 | abcam | ab-3526 | rabbit |
| p-HSL | WB | 1:1000 | cell signaling | 8334S | rabbit |
| HSL | WB | 1:1000 | cell signaling | 8334S | rabbit |
| ATGL | WB | 1:1000 | cell signaling | 2138S | rabbit |
| UCP1 | WB | 1:1000 | abcam | ab-10983 | rabbit |
| α-Tubulin | WB | 1:1000 | sigma | T5168-2ML | Mouse |

IF: Immunofluorescence

WB: Western blot

**Table S2. List of primer sequence used for Q-PCR**

| **Gene** | **Mouse primer sequences** | **Reference** |
| --- | --- | --- |
| 36B4 | Forward 5’-CACTGGTCTAGGACCCGAGAAG -3’ | Bioneer |
|  | Reverse 5’-GGTGCCTCTGGAGATTTTCG-3’ |  |
| PMP70 | Forward 5’- gattgaagagtggggtcgag -3’ | Bioneer |
|  | Reverse 5’- tctgtcgccttcccaaag -3’ |  |
| Catalase | Forward 5’- ccttcaagttggttaatgcaga -3’ | Bioneer |
|  | Reverse 5’- caagtttttgatgccctggt -3’ |  |
| DBP | Forward 5’- gggagcagtacttggagctg-3’ | Bioneer |
|  | Reverse 5’- tcagcaataactgcttcacatttt -3’ |  |
| PEX5 | Forward 5’- cacatccgcttcctatgaca-3’ | Bioneer |
|  | Reverse 5’- aaaaggctgagggtggtca-3’ |  |
| GSTA1 | Forward 5’- cttctgacccctttccctct-3’ | Bioneer |
|  | Reverse 5’- gctgccaggctgtaggaac-3’ |  |
| ACC-1 | Forward 5’- TGGACAGACTGATCGCAGAGAAAG-3’ | Bioneer |
|  | Reverse 5’- TGGAGAGCCCCACACACA-3’ |  |
| FAS | Forward 5’-GCTGCGGAAACTTCAGGAAAT-3’  Reverse 5’-AGAGACGTGTCACTCCTGGACTT-3’ | Bioneer |
| CD36 | Forward 5’-GGAACTGTGGGCTCATTGC-3’  Reverse 5’-CATGAGAATGCCTCCAAACAC-3’ | Bioneer |
| SCD-1 | Forward 5’-CCGGAGACCCCTTAGATCGA-3’  Reverse 5’- TAGCCTGTAAAAGATTTCTGCAAACC-3’ | Bioneer |
| TNFα | Forward 5′-CTGAGGTCAATCTGCCCAAGTAC-3′  Reverse 5′-CTTCACAGAGCAATGACTCCAAAG-3′ | Bioneer |
| IL-1β | Forward 5′-TCTTTGAAGTTGACGGACCC-3′  Reverse 5′-TGAGTGATACTGCCTGCCTG-3 | Bioneer |
| LPL | Forward 5’-ctggtgggaaatgatgtgg-3’  Reverse 5’-ctggtgggaaatgatgtgg-3’ | Bioneer |
| PPARα | Forward 5’-ACAAGGCCTCAGGGTACCA-3’  Reverse 5’-GCCGAAAGAAGCCCTTACAG-3’ | Bioneer |
| SREBP-1c | Forward 5’- GGAGCCATGGATTGCACATT-3’ | Bioneer |
|  | Reverse 5’- GGCCCGGGAAGTCACTGT -3’ |  |
| PGC-1α | Forward 5’-AACCACACCCACAGGATCAGA-3’ | Bioneer |
|  | Reverse 5’- TCTTCGCTTTATTGCTCCATGA-3’ |  |
|  |  |  |
| UCP1 | Forward 5’-GAGGTGTGGCAGTGTTCATTG-3’ | Bioneer |
|  | Reverse 5’- GGCTTGCATTCTGACCTTCA-3’ |  |
|  |  |  |
